# Efficient metal-free organic room temperature phosphors

**DOI:** 10.1039/d1sc00446h

**Published:** 2021-03-04

**Authors:** Aakash D. Nidhankar, Goudappagouda, Vivek C. Wakchaure, Sukumaran Santhosh Babu

**Affiliations:** Organic Chemistry Division, National Chemical Laboratory (CSIR-NCL) Dr Homi Bhabha Road Pune-411008 India sb.sukumaran@ncl.res.in; Academy of Scientific and Innovative Research (AcSIR) Ghaziabad-201002 India

## Abstract

An innovative transformation of organic luminescent materials in recent years has realised the exciting research area of ultralong room-temperature phosphorescence. Here the credit for the advancements goes to the rational design of new organic phosphors. The continuous effort in the area has yielded wide varieties of metal-free organic systems capable of extending the lifetime to several seconds under ambient conditions with high quantum yield and attractive afterglow properties. The various strategies adopted in the past decade to manipulate the fate of triplet excitons suggest a bright future for this class of materials. To analyze the underlying processes in detail, we have chosen high performing organic triplet emitters that utilized the best possible ways to achieve a lifetime above one second along with impressive quantum yield and afterglow properties. Such a case study describing different classes of metal-free organic phosphors and strategies adopted for the efficient management of triplet excitons will stimulate the development of better candidates for futuristic applications. This Perspective discusses the phosphorescence features of single- and multi-component crystalline assemblies, host–guest assemblies, polymers, and polymer-based systems under various classes of molecules. The various applications of the organic phosphors, along with future perspectives, are also highlighted.

## Abstract 2

A summary of the extremely efficient organic phosphors that utilized the best possible ways to manipulate the fate of triplet excitons for achieving a long lifetime along with impressive quantum yield and afterglow properties is provided.

## Graphical abstract

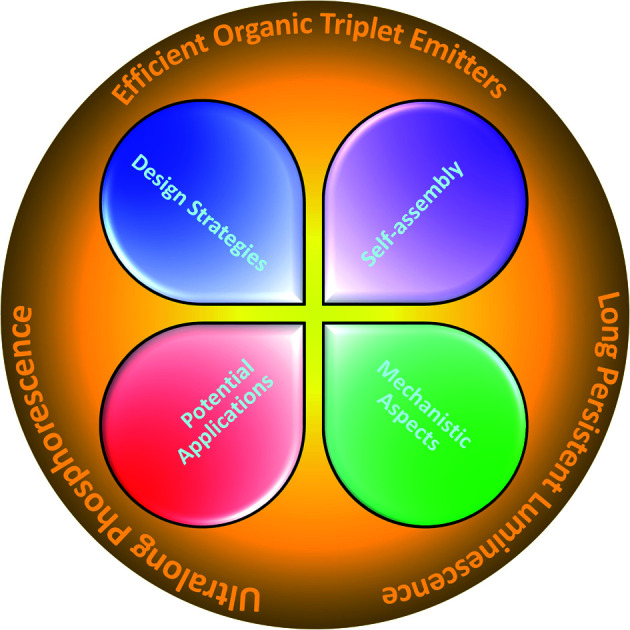

## 1. Introduction

**Fig. 1:**
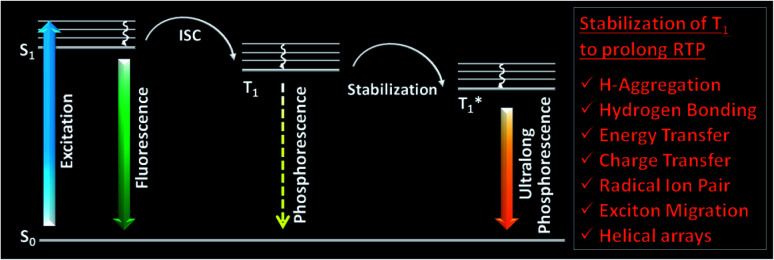
Schematic of stabilization of the triplet state, leading to ultralong phosphorescence in organic molecules.

One of the areas where organic molecules excelled in the recent past is room temperature phosphorescence (RTP).^1–30^ RTP organic molecules have attracted scientific interest due to the large Stokes shift, long lifetime, and strong afterglow that enable applications in bio-imaging, organic optoelectronics, anti-counterfeiting, sensing, *etc.*^24,25,33,34^ For the past many decades, heavy metal complexes were explored as phosphors because of the presence of ligand–metal and metal–ligand charge transfer (CT) and strong spin–orbit coupling, which improves the intersystem crossing (ISC) and controls the triplet decay rate.^27^ Despite many advantages, organometallic complexes have the drawbacks of high toxicity, limited resources, and high cost. Hence this situation promoted the search for metal-free organic molecules as alternative phosphors. However, for organic materials, the nonradiative (*k*_nr_) and quenching (*k*_q_) rates of the triplet states are much larger than the radiative decay rate (*k*_p_) under ambient conditions. Moreover, the triplet states are vulnerable to quenching by molecular oxygen, and hence RTP from metal-free organic molecules under ambient conditions continues to be challenging.^1^ Nonetheless, the recent past has witnessed a massive jump in the exploration of metal-free organic phosphors through innovative molecular designs and control over the radiative and nonradiative decay processes associated with triplet excitons. The critical parameters that are being considered while designing organic RTP (ORTP) molecules include (1) populating the triplet state by efficient singlet-to-triplet ISC, (2) minimizing the nonradiative relaxation pathways, and (3) delaying the radiative decay. In accordance with that, heteroatoms such as N, O, S, Te, and halogens are incorporated as an integral part of the molecular design of organic phosphors.^4–27^ Besides, nonradiative relaxation pathways are controlled by molecular packing in the crystalline state and with the help of supportive media such as polymers and cavitands. The compact molecular packing with the assistance of various intermolecular interactions provided extra stabilization of the triplet excitons to extend the lifetimes beyond seconds. In this way, a synergistic effect of all the supportive features resulted in enhanced RTP of metal-free organic phosphors.^5–9^ Besides, stabilization of the triplet excitons is found to be a conceptually new and exciting strategy to achieve newer heights for RTPs (Fig. 1).^20^ Many new concepts such as suppression of the nonradiative deactivation pathways of the triplet state through hydrogen bonding, H-aggregation, helical arrays, and excited-state manipulations such as CT, energy transfer (ET), radical ion pair formation, energy migration, *etc.* have been introduced to stabilize the triplet state.^20,21^

Until now, many attempts to understand the relationship between ultralong RTP (URTP) and molecular packing have been reported.^8–10^ Those studies uncovered the vital role of ordered molecular arrays in tailoring lifetime,^14–16^ emission efficiency,^18^ luminescent colour,^19^ and even realizing the unique dynamic URTP features. Even though the structure–property correlation of organic phosphors has been achieved to a certain extent, the involvement of complex factors limits a complete understanding of the underlying mechanism of URTP. Hence a deeper understanding of the supportive role of adequate molecular packing, rigidification by polymers and hosts, and related controlling factors is of great significance. Even though URTP is a fascinating concept, a slow radiative decay leading to a long-lived lifetime is not supportive for any device applications, especially in light-emitting diodes (LEDs).^31,32^ However, compared to short-lived fluorescent materials, the advantages of an ultralong lifetime and large Stokes shifts make URTP materials promising candidates for applications such as bioimaging, information storage, data encryption, anti-counterfeiting, sensing, and photodynamic therapy.^24,25,33,34^ Besides, the most critical aspect of URTP is that the triplet state excitons are susceptible to deactivation by molecular motions, oxygen, and humidity.^1,3,35^ Hence it has been extremely challenging to prolong the phosphorescent emission of organic materials at room temperature by overcoming all these hurdles. In this context, URTP of small molecule-based metal-free ORTPs has prime importance as a fundamental challenge. The numerous design strategies and self-assembly methods have been found successful in achieving an ultralong lifetime. Here, we firmly believe that a case study describing the different classes of organic phosphors and how to manage the triplet excitons to prolong the lifetime will inspire us to design better candidates for futuristic applications.

Recently, many reviews have appeared on RTP as an update of the field.^20–25,29,30^ However, the present study will mainly focus on the strategies adopted to achieve high performing metal-free organic triplet emitters, including phosphors having lifetimes of more than one second, high quantum yields, and attractive afterglow properties. More importantly, we have provided a comparison of the influence of radiative and nonradiative decay rates on the lifetime and quantum yield of each category of phosphors. Besides, the applications of ORTPs, along with future perspectives, are highlighted at the end.

## 2. Ways to improve RTP lifetime and quantum yield

The main challenges in metal-free ORTPs include weak spin–orbit coupling (SOC) (<0.1 cm^−1^) resulting in inefficient ISC, enhanced *k*_nr_ due to many deactivation pathways, and the susceptibility of the T_1_ state to oxygen and temperature.^1,3,35^ Hence, to design excellent ORTPs and estimate the performance, the following discussion can be helpful. The important parameters, *i.e.* quantum efficiency of ISC (*ϕ*_isc_) (from S_1_ to T_1_), quantum yield (*ϕ*_p_), lifetime (*τ*_p_), and radiative decay (*k*_p_), of phosphorescence can be defined as

1 *ϕ*_isc_ = *k*_isc_/(*k*_f_ + *k*_ic_ + *k*_isc_)

2 *ϕ*_p_ = *ϕ*_isc_*k*_p_*τ*_p_

3 *τ*_p_ = 1/*k*_p_ + *k*_nr_

4 *k*_p_ = (64π^4^/3*h*^4^*c*^2^)Δ*E*^3^_T_1_→S_0__∣*μ*_T_1_→S_0__∣^2^

where *k* denotes the rates of the singlet emission process such as *k*_f_, *k*_ic_ and *k*_isc_ related to the fluorescence (from S_1_ to S_0_), internal conversion (IC) (from S_*n*_ to S_1_) and ISC (from S_1_ to T_1_), respectively. At the same time, the rates related to the triplet state are denoted as *k*_p_ and *k*_nr_ for radiative and nonradiative decays (from T_1_ to S_0_), respectively. It has to be noted that all these rate parameters related to singlet and triplet transitions completely control the characteristic phosphorescence features of pure ORTPs. An in-depth analysis of eqn (1)–(3) reveals that an enhanced *k*_isc_, a reduced *k*_nr_, and a slow *k*_p_ are essential for an improved RTP lifetime. Another critical parameter not discussed much is the triplet quenching rate *k*_q_ which can be minimized by rigidification of the phosphor using a host or polymer support.^20,29,30^ Besides, eqn (1)–(3) indicate that conditions such as enhanced *ϕ*_isc_ and *k*_p_ > *k*_nr_ are required to improve *ϕ*_p_. However, to achieve long *τ*_p_ a reduction of both *k*_p_ and *k*_nr_ is needed. Hence the impact of *k*_p_ is ultimate in achieving high *ϕ*_p_ and long *τ*_p_. Eqn (4) shows that an increment in the singlet–triplet energy gap Δ*E*_T_1_→S_0__ will accelerate *k*_p_ and eventually reduce *k*_nr_. The above two conditions related to *k*_p_ are contradictory when looking for a long lifetime and a high quantum efficiency. It indicates that simultaneous enhancement of both *ϕ*_p_ and *τ*_p_ is challenging. The below sections describe the various parameters controlling the RTP features.

### 2.1. Rate of intersystem crossing *k*_isc_

There have been several attempts to obtain an enhanced *k*_isc_ necessary for efficient RTP, such as effective SOC and small Δ*E*_ST_.^36–38^ In this direction, the major directive for a high *k*_isc_ through effective SOC has come from El-Sayed's rule, which states that effective orbital overlapping is possible in a singlet to triplet transition with different molecular–orbital configurations.^39^ In other words, compared to the transition from ^1^(n,π*) to ^3^(n,π*) or from ^1^(π,π*) to ^3^(π,π*), effective ISC can be observed in transitions from ^1^(n,π*) to ^3^(π,π*) or from ^1^(π,π*) to ^3^(n,π*) states. Many recent successful designs showed that the presence of n orbitals perpendicular to π orbitals is beneficial to facilitate a strong SOC and thereby promote the ISC from singlet to triplet excited states.^12,14^ Hence the presence of hybrid (n,π*) and (π,π*) configurations is supportive for URTP. An enhanced *k*_isc_ through SOC significantly contributed to achieve high *ϕ*_p_. However, a long *τ*_p_ heavily depends on the stabilization of triplet excitons through multiple pathways.

### 2.2. Energy gap Δ*E*_ST_

One of the reasons for the inefficient RTP in organic molecules is the large Δ*E*_ST_ due to inappropriate molecular designs. It has been noticed that *k*_isc_ can be significantly promoted by small Δ*E*_ST_, which depends on the spatial overlap of the highest occupied molecular orbital (HOMO) and lowest unoccupied molecular orbital (LUMO) wavefunctions. A strong CT interaction between the donor and acceptor building units of a phosphor can be influential because CT interaction can control the spatial overlap. A CT state can be introduced either by bridging donor–acceptor (D–A) groups or through the assistance of intermolecular interactions between donor and acceptor molecules. Such examples for enhanced RTP through small Δ*E*_ST_ driven by the CT state^38^ and through aggregation controlled management of Δ*E*_ST_ (ref. 40) are available in the literature. The formation of various aggregates can dictate Δ*E*_ST_ values and thereby influence ISC.

### 2.3. Rate of nonradiative decay *k*_nr_

The current era of organic phosphors and the sudden developments are attributed to crystallization to a greater extent. The crystallization of phosphors using intermolecular interactions such as π–π stacking, van der Waals, halogen, and hydrogen bonding suppresses the nonradiative decay pathways to facilitate RTP.^41^ Besides, host–guest assemblies, immobilization of phosphors in frameworks, and polymers also protected the phosphors from nonradiative decay. In this case, the most celebrated “H-aggregation” was found to be effective in stabilizing the triplet excitons to achieve long-lived RTP.^42^ As per eqn (2) and (3), a variation in *k*_nr_ also significantly alters the values of both *ϕ*_p_ and *τ*_p_. Hence the design strategies should consider the incorporation of corresponding functional moieties to impart various noncovalent interactions to regulate *k*_nr_. Another vital strategy adopted to reduce *k*_nr_ by decreasing reorganization energy is deuteration, which blocks the molecular vibrations.^43^ The high Δ*E*_T_1_→S_0__ value was also found to be effective in suppressing the nonradiative decay.^2,3,39^

### 2.4. Rate of radiative decay *k*_p_

Eqn (4) shows that *k*_p_ is complicated and mainly determined by many factors involving both the singlet and triplet states, such as SOC, transition dipole moment *μ*_T_1_→S_0__, and energy gap Δ*E*_T_1_→S_0__. A high Δ*E*_T_1_→S_0__ accelerates *k*_p_ to facilitate RTP with a reduced lifetime and it is the same with the transition dipole moment as well.^2,3,39^ It has been found that in ORTPs showing excellent features, an exceptional stabilization of the triplet excitons through various supports delays the radiative decay. In such systems, *k*_p_ is suppressed to achieve a long lifetime over seconds. A slow *k*_p_, in turn, enhances *τ*_p_ to realize URTP, while it inversely affects *ϕ*_p_.

### 2.5. Rate of quenching *k*_q_

Along with other factors controlling the efficiency of RTP, the suppression of *k*_q_ is also equally important. Even though recent studies have employed crystalline assemblies and the support of polymers, hosts, and porous structures to avoid the effect of moisture and oxygen, the quenching rate of the triplet excitons is found to be crucial to deliver excellent RTP features under ambient conditions. Hence the deactivation patterns of the triplet excitons require more attention. The current studies on ORTPs lack a deeper understanding of the effect of *k*_q_, assuming that stabilization through self-assembly or other supports will control *k*_q_.^20^

### 2.6. Triplet exciton diffusion

Recent studies have shown the importance of triplet exciton diffusion length on persistent RTP.^44^ It has been suggested that short-range triplet exciton diffusion can suppress *k*_q_ by stopping the excitons from reaching the trap sites. The importance of exciton diffusion length is more dominant in the case of crystalline arrays with more traps. However, a very recent demonstration indicated that the triplet exciton diffusion with the assistance of helical arrays of phosphor played an essential role in delaying the triplet radiative decay to extend lifetime beyond 4 s.^45^ Hence the quality of the generated crystals or thin films and management of triplet excitons can impart excellent RTP features. More studies in this direction are required for detailed understanding and further development. In addition, hyperfine-coupling (HFC) driven intersystem crossing in CT complexes and radical ion pairs (RIPs),^46^ singlet fission,^47^*etc.* were also found to be useful in controlling the RTP features.

The above sections describe the possible ways for organic phosphors to achieve enhanced RTP features. The implemented molecular designs have been found to be successful to a certain extent and thus delivered some exceptional RTP candidates exhibiting a lifetime longer than one second. In this Perspective, we attempt to reveal the special effects in molecular design and ground state arrangements of metal-free organic phosphors to exhibit prodigious RTP qualities. The recent progress in the lifetime, quantum yield, and afterglow properties of these unusual candidates promoted metal-free ORTPs as a capable material in many functional applications. We want to mention that the dedicated efforts of various research groups worldwide made significant contributions in this area to gain an admirable position for ORTPs in current research.^1–30^

## 3. Efficient organic phosphors

**Scheme 1:**
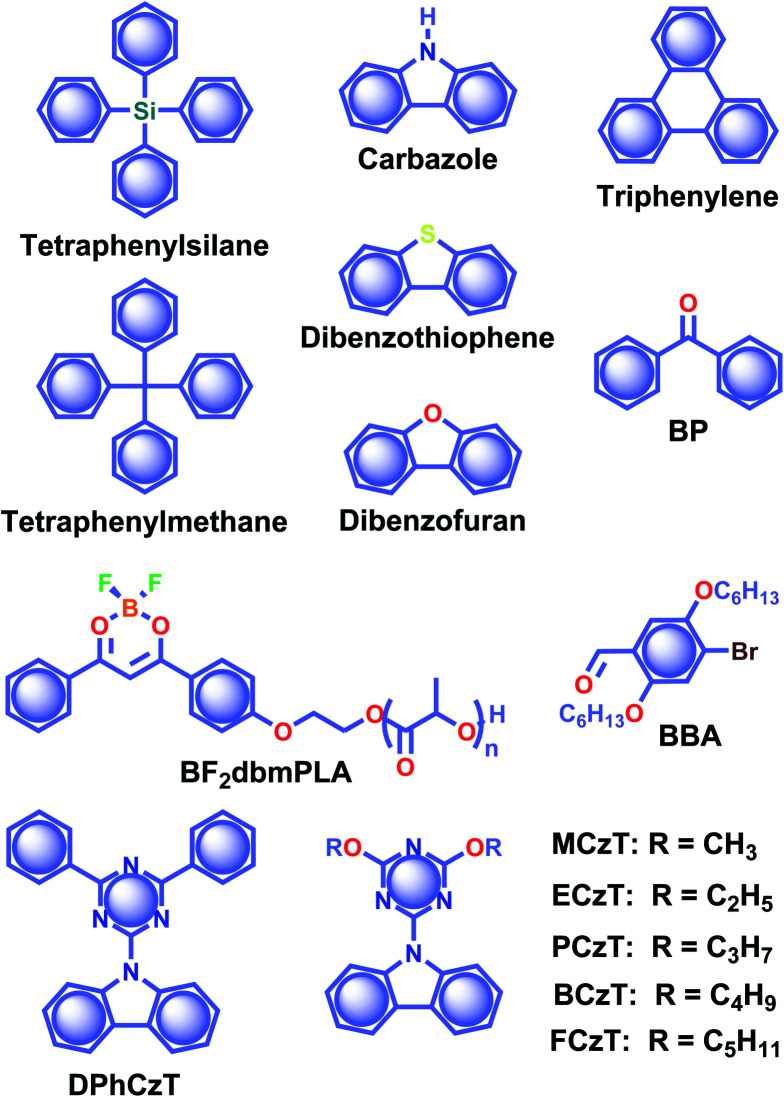
Chemical structure of phosphors tetraphenylsilane, tetraphenylmethane, carbazole, dibenzothiophene, dibenzofuran, triphenylene, **BP**, **BF2dbmPLA**, **BBA**, **DPhCzT**, **MCzT**, **ECzTPCzT**, **BCzT**, and **FCzT**.

RTP of metal-free organic molecules fascinated the scientific community as early as the 1940s, and the exciting demonstrations in the early stages have been summarised in many reviews.^39,48–50^ The initial experiments were limited in solutions, that too under cryogenic and oxygen-free conditions.^51–54^ Later, the developments were focused on RTP of salts of aromatic carboxylic acids, phenols, amines, and sulfonic acids in rigid media including boric acid glass,^52–54^ and filter paper, silica or alumina,^55–59^ micelles,^60^ and hosts such as cyclodextrins,^61^ zeolites,^62^ hemicarcerand,^63^*etc.* The rigid matrices prepared using glucose, sucrose, citric acid, and tartaric acid have supported the RTP of organic molecules.^51,64^ Similarly, plastics such as poly(methylmethacrylate) and poly(vinyl alcohol) also provided a rigid medium for organic phosphors to excel.^65–67^ The subsequent investigations on ORTP over a few decades emerged the area and found it useful for many potential applications.^68,69^ In between, RTP of crystals was reported in 1939 by Clapp and coworkers.^70^ Crystals of tetraphenylsilane and tetraphenylmethane (Scheme 1) showed RTP afterglow emission up to 23 s visible to the bare eye. Later, in 1978, Bilen and coworkers studied the afterglow of carbazole, dibenzothiophene, dibenzofuran, triphenylene (Scheme 1), *etc.*, having a lifetime up to 4.85 s in the crystal state.^71^ After a long gap, the technological improvement in materials chemistry during the last decade resulted in a drastic development in RTP of metal-free organic molecules.^20–25,29,30^ Many new molecular designs and self-assembly models have been experimented with and eventually URTP was realized. The following sections detail the recent advancements of ORTPs.

In 2007, the research group of Fraser came up with boron difluoride dibenzoylmethane (BF_2_dbm) conjugated poly(lactic acid) **BF2dbmPLA** (Scheme 1) having oxygen-sensitive RTP.^72^ Later in 2010, Tang and coworkers reported RTP of a series of benzophenone **BP** derivatives (Scheme 1) and found that the presence of a carbonyl group or halogen atom, and nonplanar conformation are supportive factors in RTP.^73^ RTP of 2,5-dihexyloxy-4-bromobenzaldehyde **BBA** (Scheme 1) was reported by Kim and coworkers in 2011.^74^ This molecule shows weak fluorescence in solution; however, it exhibits a green phosphorescence emission with a lifetime of 5.4 ms in the crystal state. Single-crystal XRD analysis revealed that RTP is due to the presence of close contact between the bromine and the carbonyl oxygen of the neighbouring molecule (C=O⋯Br–C, 2.86 Å). RTP retains a lifetime longer than 100 ms with an afterglow approaching seconds after cessation of excitation. The above three studies greatly attracted the attention towards organic phosphors and hence resulted in a visible change in the area.

### 3.1 Single and two-component crystalline organic phosphors

Recently, significant developments have occurred in single-component crystalline assemblies that exhibit long *τ*_p_ along with high *ϕ*_p_.^75^ In general, RTP of such single-component assemblies is enhanced by crystallization mainly because of the following reasons: (1) availability of specific intermolecular interaction in the crystal state to improve ISC through SOC; (2) intact molecular packing suppresses the molecular motions and eventually helps to minimize *k*_nr_ of triplet excitons, (3) crystalline assemblies will provide protection from triplet quenching by oxygen. The recent exciting development of URTPs is strongly supported by molecular organization in the crystal state, which stabilizes the excited triplet state by trapping triplet excitons and controls both radiative and nonradiative decays.

In 2015, Chen, Liu, Huang, and coworkers synthesized a series of pure organic molecules of carbazole and triazine containing O, N, and P atoms (Scheme 1).^42^ Notably, the presence of heteroatoms facilitates the spin-forbidden transfer of singlet-to-triplet excited states through n–π* transitions to populate triplet excitons. In this series, 9-(4,6-diphenyl-1,3,5-triazin-2-yl)-9*H*-carbazole **DPhCzT** and 9-(4,6-diethoxy-1,3,5-triazin-2-yl)-9*H*-carbazole **ECzT** (Scheme 1) exhibited *τ*_p_ up to 1.35 s and 1.05 s, respectively. The enhanced ISC in these molecules is due to the increased number of energy transition channels supported by the H-aggregated dimers of **DPhCzT** and **ECzT** in the crystals. The strong coupling *via* π–π stacking in the H-aggregate dimers with a large transition dipole moment provides stabilization and thus protects the triplet excitons. The stabilized triplet excited state 
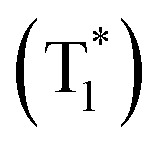
 functions as an energy trap at a lower energy level, offering suppressed radiative and nonradiative deactivation decay rates in favour of long-lived phosphorescence. As a continuation, by varying the alkyl chain length on the triazine unit, the same group achieved photoactivation assisted smart URTP materials.^76^ Among the molecules, the lifetime of **BCzT** (Scheme 1) drastically increased from 1.8 ms to 1.33 s upon photoirradiation for 10 min. The prolonged irradiation of molecules with UV light suppresses *k*_nr_ by controlling the molecular motion to enhance both RTP emission intensity and lifetime. A significant difference in the distances of intermolecular interactions between adjacent molecules is observed after photoactivation. As the length of the alkoxy chain increased, both the lifetime of photoactivation and deactivation for URTP decreased drastically from **MCzT** to **FCzT**. This study points to the importance of photo-irradiation assisted control of nonradiative transition in ORTPs.

**Scheme 2:**
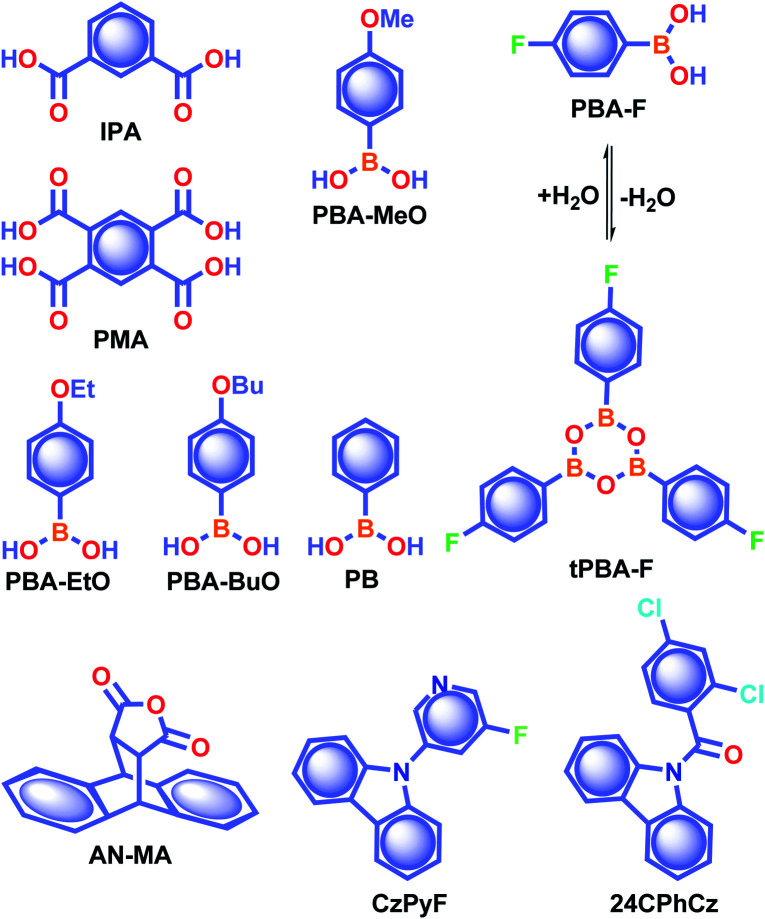
Chemical structure of benzoic acids **IPA** and **PMA**, boron-containing phosphors **PBA-MeO**, **PBA-EtO**, **PBA-BuO**, **PBA-F**, **tPBA-F**, and **PB**, and **AN-MA**, **CzPy**, and **24CPhCz**.

In 2015, Yuasa and his team explained the nuclear spin magnetism-assisted spin-exchange of a radical ion pair (RIP).^46^ In this study, the RTP afterglow of benzoic acid derivatives such as isophthalic acid **IPA** and pyromellitic acid **PMA** (Scheme 2) was observed for several seconds. Phosphorescence measurements of **IPA** and **PMA** revealed the presence of bands at 532 and 533 nm with 0.970 and 1.1 s lifetime, respectively. Photoexcitation of these carboxylic acid derivatives leads to the generation of singlet and further triplet RIPs through hyperfine-coupling (HFC) induced spin conversion, mediated through the magnetic field of neighbouring ^1^H nuclear spins. Here, deuterium labeling of carboxylic acids suppressed the phosphorescence intensity, confirming the HFC mechanism and a nuclear spin magnetism-assisted spin conversion (^1^RIP–^3^RIP) responsible for URTP. In another report, Yan and coworkers reported the phosphorescence lifetime of **IPA** in the crystal state as 1.11 s and explained that the presence of only one type of hydrogen bonding interaction in **IPA** stabilizes the carboxylic acid dimers.^77^

**Fig. 2:**
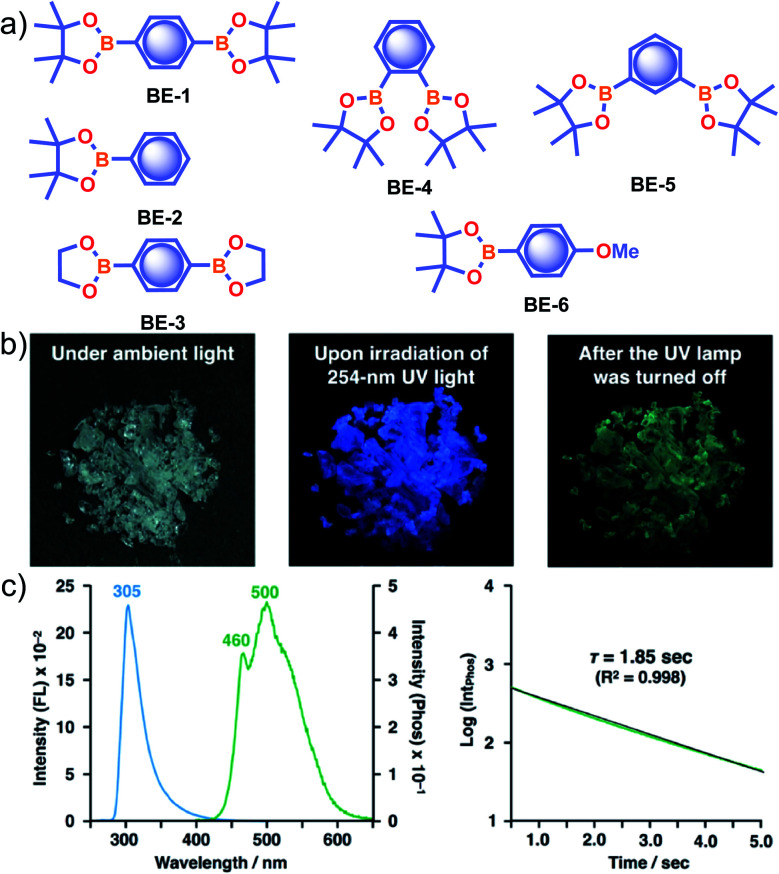
(a) Chemical structure of aryl boronic esters **BE-1–6**. (b) Photographs of **BE-1** under ambient light (left) and irradiation with 254 nm UV light in the dark (middle), and after the UV light was turned off in the dark (right). (c) Fluorescence and phosphorescence spectra (left) and the corresponding phosphorescence decay profile of **BE-1** (right). Reproduced with permission from ref. 78. Copyright 2017, American Chemical Society.

Recently, many aryl boronic acids and esters with stable and extended RTP have been reported. In one of the first reports, Nakai, Fukushima, and coworkers reported long-lived RTP of a series of aryl boronic esters **BE-1–5** (Fig. 2a).^78^**BE-1** displayed RTP in the solid-state with a green afterglow that lasted for several seconds (Fig. 2b and c). In this series **BE-1–6**, the value of *τ*_p_ varied as 1.85, 1.79, 1.65, 1.73, 1.57, and 1.39 s, respectively (Fig. 2c). A combined experimental and theoretical study revealed that an out-of-plane distortion is introduced at the (pinacol) B–C_ipso_ moiety in the 
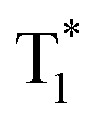
 state, and it enables the mixing of π and σ orbitals to enhance SOC and thereby lead to URTP. Later, Li and coworkers studied the RTP of many commercially available phenylboronic acids and their thermally prepared triphenylborazine derivatives.^41^ The phosphorescence spectrum of 4-methoxyphenyl boronic acid **PBA-MeO** (Scheme 2) revealed two resolved emission peaks at 457 and 488 nm with *τ*_p_ of 2.24 and 2.19 s, respectively. The long lifetime is due to rigid conformation and strong intermolecular interactions *via* hydrogen bonds, which decrease *k*_nr_. An effective π–π stacking stabilizing the triplet excitons also contributes to bright and prolonged RTP. The importance of π–π stacking interactions was further analyzed by theoretical calculations of dimers, indicating that strong π–π stacking decreases Δ*E*_ST_ to favour ISC. The authors studied RTP by varying the length of the alkyl chain and found that **PBA-EtO** and **PBA-BuO** also showed RTP with *τ*_p_ of 1.11 s and 1.28 s, respectively. In this series of boronic acids, the support of H-bonds through C–H⋯F interactions enabled **PBA-F** and the thermally prepared triphenylborazine **tPBA-F** derivative (Scheme 2) to show a long lifetime of 1.34 and 1.96 s, respectively.

**Fig. 3:**
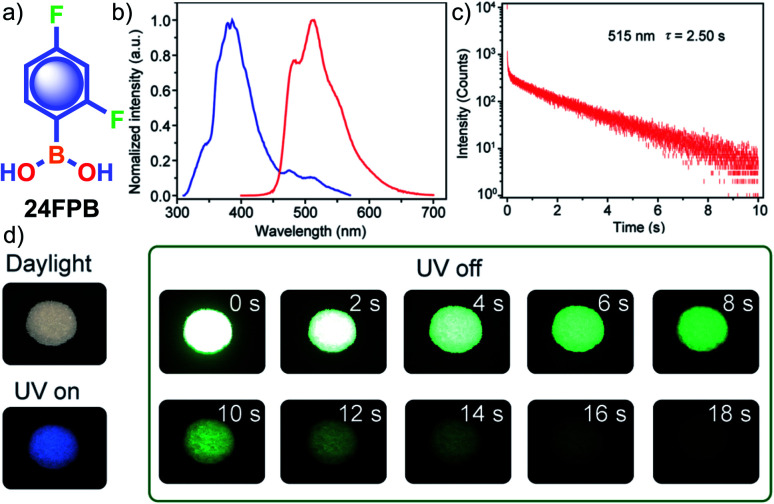
(a) Chemical structure of **24FPB** and (b) steady-state photoluminescence (blue line) and phosphorescence (red line) spectra of **24FPB**. (c) Phosphorescence decay profile of the emission band at 515 nm. (d) Photographs of the **24FPB** crystal taken at different time intervals before and after removing the excitation source (a 365 nm UV lamp). Reproduced with permission from ref. 80. Copyright 2019, WILEY-VCH.

In the same year, HFC driven ISC in CT complexes was reported by Yuasa and coworkers using phenylboronic acid derivatives (**PBs**) such as phenyl-mono-boronic acid **PB** (Scheme 2) and *p*-phenylenediboronic acid ethyleneglycol ester **BE-3** (Fig. 2a) as examples.^79^ The phosphorescence intensities of **PBs** mainly depend on the magnetic-field and spin-isotope effects controlled by HFC. Interestingly, **PB** showed a RT afterglow for several seconds with *τ*_p_ of 1.2 s. Furthermore, the authors investigated the effect of steric bulkiness on phosphorescence by taking **BE-3** as an example, which showed a pale blue afterglow for about 12 s with *τ*_p_ of 1.6 s. The luminescence quantum yield of **PB** and **BE-1** was found to be 18 and 77%, respectively. The phosphorescence mechanism was summarized to follow the transitions S_0_ → ^1^CT → ^3^CT → T_1_ → S_0_, indicating the direct involvement of both singlet and triplet CT states. Furthermore, studies on the effect of halogen on RTP of **PBs** showed that among the series, only 2-(4-fluorophenyl)-1,3,2-dioxaborolane **PBA-F** exhibited long *τ*_p_ of 1.7 s. In this line, Huang and his group enhanced *τ*_p_ by introducing multiple fluorine atoms on the phenylboronic acid **24FPB** (Fig. 3a and b).^80^ The maximum lifetime of 2.50 s was exhibited by 2,4-difluorophenylboronic acid **24FPB** crystals (Fig. 3c). The prolonged lifetime is because of the stabilization of the triplet excited state by H-aggregation and intramolecular O–H⋯F hydrogen bonding (2.22 Å) in the crystal. The hydrogen bonding in crystals fixed the dihedral angle (*Θ*) between the benzene ring and the boronic acid group, resulting in rotation confinement leading to a longer lifetime. Interestingly, a persistent green luminescence for **24FPB** was observed for more than 20 s after the UV light was turned off (Fig. 3d).

Since intermolecular interaction assisted packing in the crystalline assembly plays a critical role in ORTP, the examples of polymorphs need a special mention.^81^ Polymorphs offer the opportunity to develop crystals with varying sizes, shapes, and optical properties through tuning the cultivation conditions. It has been noticed that even slight variations in the molecular packing impart significant changes in the excited state properties. In this context, the benefit of polymorphism is the informative correlation between molecular packing and the resulting functional properties. In another way, it encourages rational molecular design to improve the optical properties and hence RTP polymorph is an interesting topic of research.

Yuan and coworkers reported a long lifetime for the cycloaddition product **AN-MA** of anthracene and maleic anhydride (Scheme 2).^82^ Out of the two polymorphs form A and form B, afterglow emission of the latter one lasted for several seconds with *τ*_p_ of 1.6 s and *ϕ*_p_ of 8%, because of its much stronger intramolecular π–π interactions. The presence of the carbonyl group and oxygen atoms with lone pairs, together with the effective intra- and intermolecular interactions, helps **AN-MA** to achieve bright green RTP emission. The relatively low *k*_nr_ value resulting from the more rigid conformations significantly contributed towards the long lifetime. Cai *et al.* reported that out of the three polymorphs formed by hydrogen-bonded frameworks of benzene-1,3,5-triyltris((9*H*-carbazol-9-yl)methanone), only one with two different types of tetragonal pore in the crystal packing exhibited URTP of 198 ms. The presence of strong intralayer π–π interactions between carbazole units in the framework stabilized the triplet excitons to achieve URTP.^83^ Similarly, Yang *et al.* demonstrated how the different molecular conformations in the polymorphs of 4-(4*a*,10*a*-dihydro-10*H*-phenothiazin-10-yl)benzonitrile control RTP.^84^ In the series of three polymorphs generated, the one with the more intermolecular interactions has the more extended RTP lifetime of 266 ms. These studies showed that the various modes of packing arising from the different molecular configurations greatly contribute to the incomparable RTP features of the polymorphs. Due to the lower number of reports and nominal performance, RTP polymorphs and the related discussion is restricted in this Perspective.

To understand the effect of a heavy atom to obtain longer RTP, Sasabe, Kido and coworkers studied the RTP features of a series of 3-pyridylcarbazole derivatives with H, F, Cl, Br, and I as substituents on the pyridine ring.^85^ The crystals of the fluorine-substituted derivative **CzPyF** (Scheme 2) showed an ultralong *τ*_p_ of 1.1 s and *ϕ*_p_ of 1.2%. Theoretical and experimental data revealed the crucial role of n orbital on the central pyridine ring in enhancing the intersystem crossing between ^1^CT* and the locally excited triplet (^3^LE*) states. X-ray crystallographic studies indicated that both the pyridine ring and fluorine atom contributed to the enhancement of RTP through restricted motion owing to weak C–H⋯N and H⋯F hydrogen-bonding interactions. The presence of a halogen atom with larger electronegativity enabled a longer RTP lifetime in this series. Similarly, in 2019, Shi, An, Huang, and coworkers provided a detailed study related to the critical role of molecular stacking in generating triplet excitons by using a series of carbazole derivatives having chlorine substitution at different positions.^86^ The combined experimental and calculated results revealed that **24CPhCz** (Scheme 2) with robust intermolecular coupling between carbazoles exhibited long *τ*_p_ of 1.06 s and *ϕ*_p_ of 2.5%. A detailed crystal structure analysis of **24CPhCz** showed that the existence of intermolecular interactions played an essential role in enhancing the lifetime. The molecules were restricted by abundant CCl⋯π, C=O⋯Cl, CCl⋯H–C, Cl⋯Cl, and C–H⋯π interactions. The restricted nonradiative transitions through molecular packing in the crystal state prolong the lifetime. However, a weak interchromophoric coupling between carbazoles resulted in weak phosphorescence for the other derivatives in the series.

**Fig. 4:**
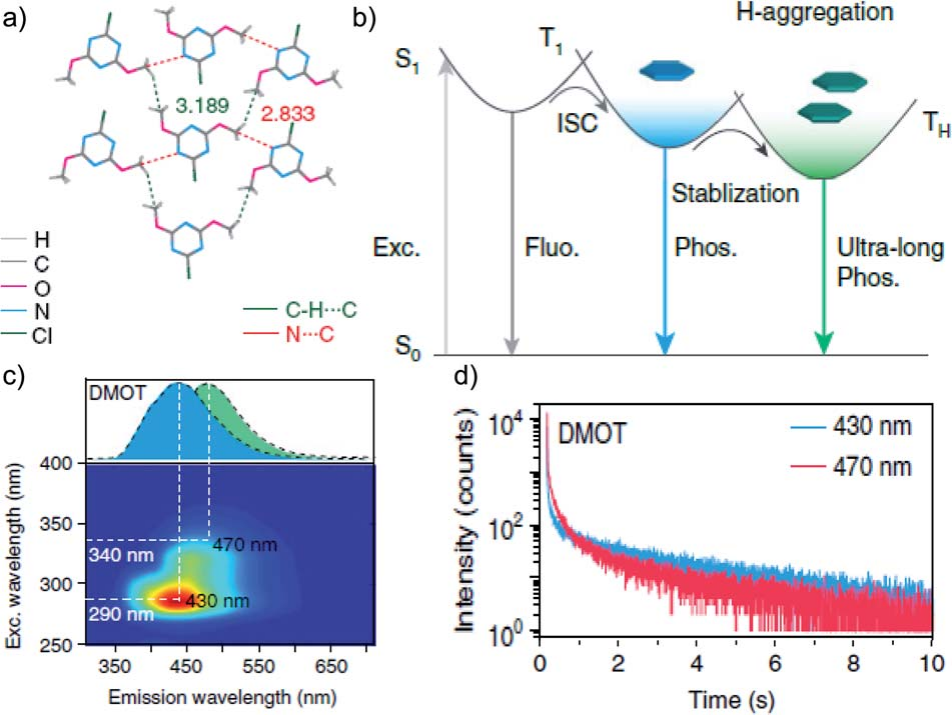
(a) Top-view crystal structure of **DMOT** showing detailed information on the intermolecular interactions. (b) Proposed mechanism and molecular design for excitation-dependent colour-tunable URTP. (c) Excitation-phosphorescence mapping of **DMOT**. (d) Phosphorescence decay profiles of emission bands at 430 and 470 nm for **DMOT**. Reproduced with permission from ref. 87. Copyright 2019, Springer Nature Limited.

Tunable emission organic phosphors are rare and are difficult to achieve in a single-component phosphor. A tunable phosphorescence under different excitation wavelengths was reported by Gu *et al.* utilizing the available multiple emitting centers in a phosphor (Fig. 4a).^87^ A triazine derivative, 2-chloro-4,6-dimethoxy-1,3,5-triazine **DMOT** (Fig. 4a and b), contains various heteroatoms that improve the *k*_isc_ to boost the population of triplet excitons. The planar structure of the molecule strongly supports H-aggregation through multiple intermolecular interactions such as N⋯C, CH⋯C, and π–π interactions with the surrounding six molecules (Fig. 4a). Besides, H-aggregation assisted restricted molecular motion in the crystal ensures excellent phosphorescence features with *τ*_p_ of 2.45 s and *ϕ*_p_ of 31.2% (Fig. 4b–d). Interestingly, upon changing the excitation wavelength from 250 to 400 nm, the emission colour of **DMOT** was tuned from violet to sky blue, owing to single-molecule and H-aggregate phosphorescence, respectively (Fig. 4c). Such tunable emission smart RTP material will be useful for displays, sensors, and imaging applications.

**Scheme 3:**
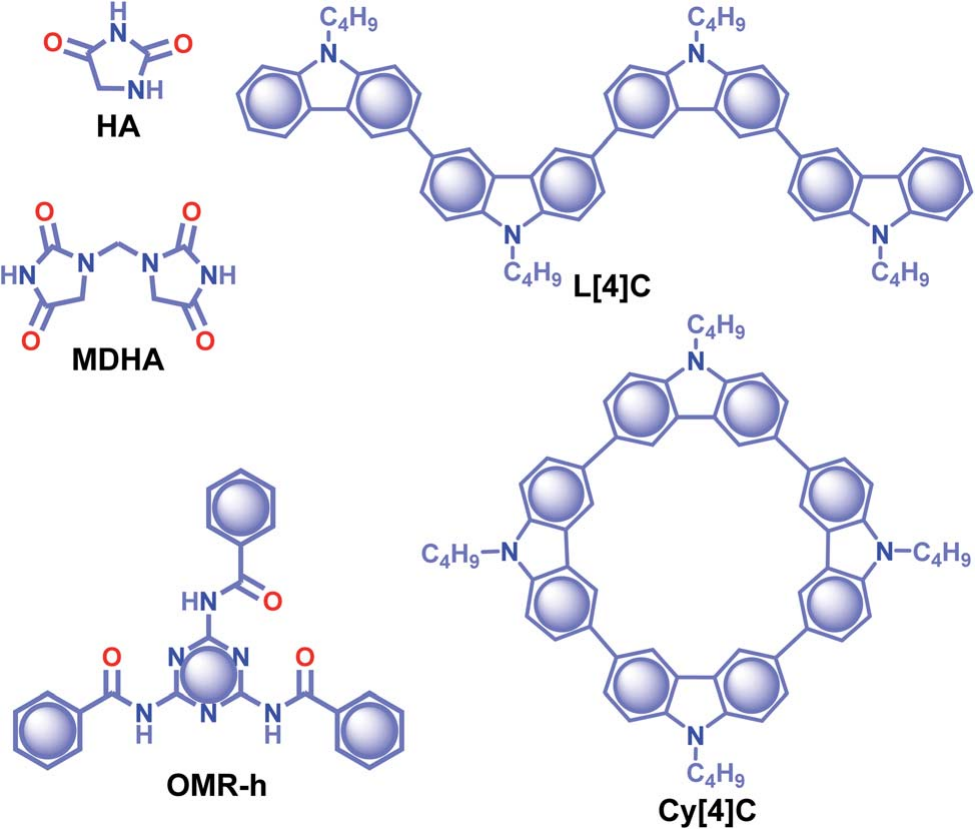
Chemical structure of **HA**, **MDHA**, **OMR-h**, **L[4]C** and **Cy[4]C**.

Similarly, Yuan and coworkers worked on an unexplored pyrimidine molecule, hydantoin (**HA**) (Scheme 3), with tunable emission colour in response to the excitation wavelength.^88^ The synergistic effect of through-space conjugation between carbonyls (C=O) and nitrogen (N) heteroatoms, and intermolecular interactions through multiple hydrogen-bonds enabled **HA** to be an excellent RTP emitter with *τ*_p_ of 1.74 s and *ϕ*_p_ up to 21.8%. Crystals of **HA** displayed sky-blue and yellowish-green afterglows lasting for over 10 s upon excitation with 312 and 365 nm UV lights, respectively. A stable self-assembled network with the adjacent molecules using multiple H-bonds, C=O⋯H and O=C⋯C=O (π–π) interactions at a relatively shorter distance strengthens the assembly and implies extended through-space delocalization. A dimer of **HA**, 1,1′-methylenedihydantoin **MDHA** (Scheme 3), also exhibited tunable URTP, but with comparatively lower efficiency having *τ*_p_ of 1.27 and *ϕ*_p_ of 3.6%. The tunable RTP feature is supported by a clustering-triggered emission mechanism, where the presence of different clusters with through-space conjugation and conformation rigidification resulted in tunable RTP.

**Fig. 5:**
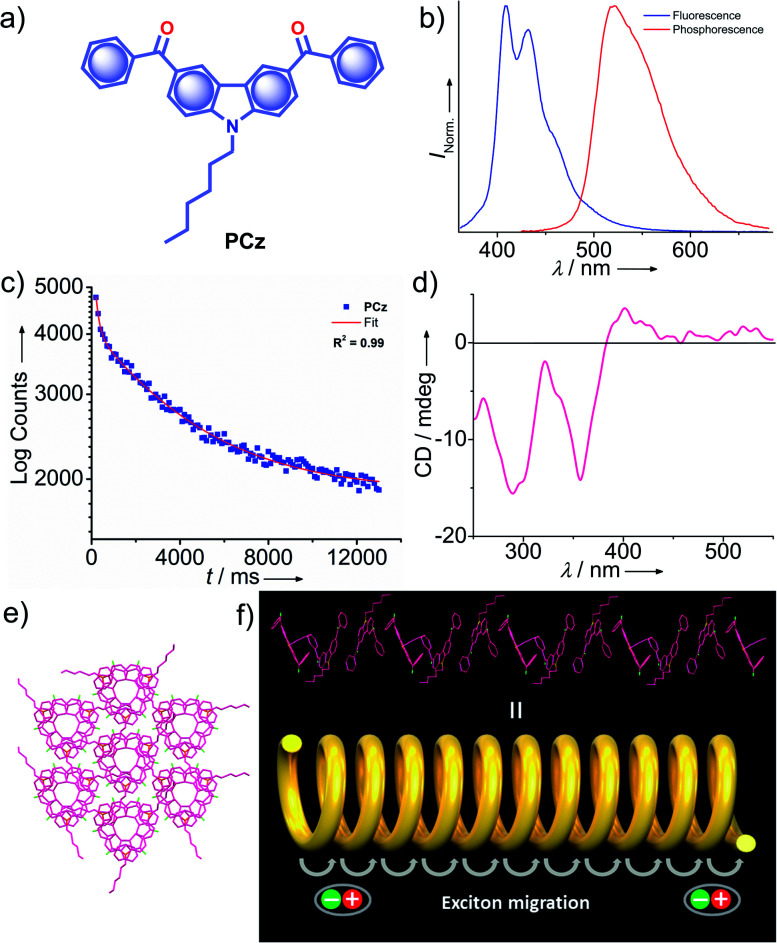
(a) Chemical structure of **PCz**. (b) Steady-state photoluminescence (blue line) in solution and phosphorescence (red line) spectra in the crystal state of **PCz**. (c) Phosphorescence decay profile of **PCz** crystals. (d) The solid-state CD spectrum of **PCz** crystals. (e) Six adjacent helical arrays of **PCz** leading to extended columnar packing in the *c*-axis. (f) Schematic of the helical array of **PCz** leading to triplet exciton migration. Reproduced with permission from ref. 45. Copyright 2020, WILEY-VCH.

Very recently, Babu and coworkers came up with a new strategy of stabilizing triplet excitons by helical molecular packing (Fig. 5).^45^ An *N*-alkylated carbazole decorated with phenylmethanone units **PCz** (Fig. 5a) exhibited URTP with high efficiency (*τ*_p_ > 4.1 s and *ϕ*_p_ of 11%) (Fig. 5b and c). A helical molecular array of **PCz** in the crystal state enabled the singlet–triplet states to be mixed up to enhance ISC. A right-handed helical molecular array of **PCz** acts as a trap and exhibits triplet exciton migration to deliver the exceptionally long lifetime (Fig. 5d and e). An extended molecular array was formed by the arrangement of molecules mainly through π–π interaction (3.34 Å) between carbazole and phenylmethanone units of adjacent molecules. The thus-formed 1D-helical array is stabilized by CH⋯π interaction between the alkyl chain on carbazole and phenylmethanone unit in the adjacent helical columns. Space filled packing rigidifies the molecular conformations and remarkably blocks the nonradiative decay pathways. A combined experimental and theoretical study sheds light on the stabilization of the triplet state by the helical arrays. The micro rods of **PCz** exhibit triplet exciton migration that prolongs RTP lifetime (Fig. 5f). In contrast to other carbazole based small molecule phosphors, **PCz** failed to show afterglow emission under ambient conditions.

In 2020, Shan and coworkers reported URTP of organic micro-rods of **OMR-h**, which is synthesized by heating a mixture of melamine and benzoic acid in an aqueous solution (Scheme 3).^89^ In the presence of water, the micro-rods form a hydrogen-bonded network that rigidifies the molecular motion. A significant enhancement in RTP features with *τ*_p_ of 1.64 s and *ϕ*_p_ of 11.4% was observed under the wet conditions. The observed lifetime of the hydrogen-bonded structure of **OMR-h** is one of the extended lifetimes of ORTP materials in water. A cyclization-driven enhancement of a less RTP active candidate, *N*-butyl carbazole (*τ*_p_ of 1.45 ms), is reported by Zhu *et al.*^90^ An increase in the conjugation resulted in an efficient ISC for both linear **L[4]C** and cyclic **Cy[4]C** derivatives of *N*-butyl carbazole (Scheme 3) and helped to achieve longer lifetimes of 2.24 and 3.41 s, respectively. The prolonged lifetime is correlated with the significantly lower Δ*E*_ST_ for **Cy[4]C** with a near-planar structure. Moreover, the synergistic effect of rigidification also contributed to suppress the nonradiative decay. The obtained value is highest among the lifetimes for an organic phosphor without any heavy atom or a carbonyl group.

**Fig. 6:**
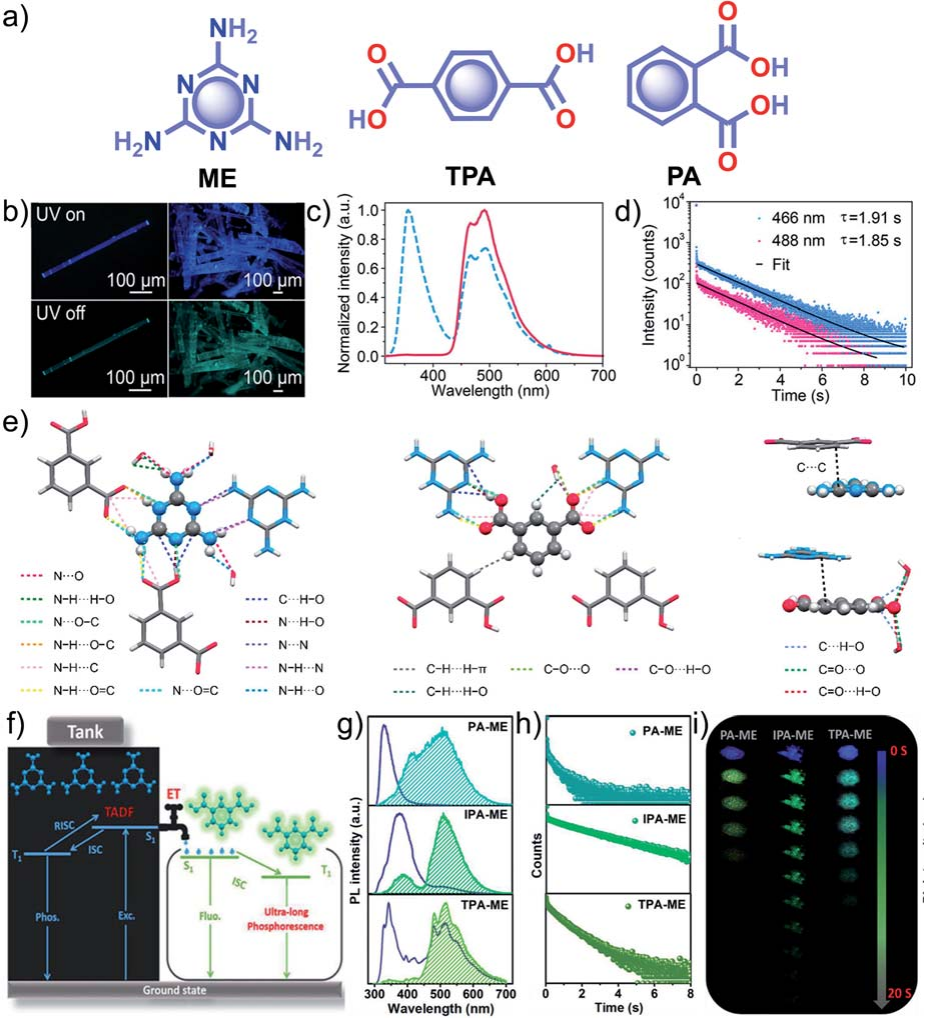
(a) Chemical structure of **ME**, **TPA** and **PA**. (b) Photographs of emission (top) and phosphorescence (bottom) of the **ME–IPA** cocrystal. (c) Steady-state photoluminescence (blue dotted line) and phosphorescence (red line) spectra along with (d) phosphorescence decay of the emission bands at 466 and 488 nm, respectively, of **ME–IPA**. (e) Molecular packing of **ME–IPA** showing intermolecular interactions from the same plane with **ME** as the center and **IPA** as the center along with **ME** and **IPA** with adjacent planes. (f) Schematic of TADF-assisted transfer from **ME** to **IPA** enhancing the RTP lifetime. (g) Prompt and delayed fluorescence spectra and corresponding (h) time-resolved fluorescence decay profiles, and (i) photographs showing the long-afterglow of **ME–PA**, **ME–IPA**, and **ME–TPA**. Reproduced with permission from (a–e) ref. 91 (copyright 2018, American Chemical Society) and (f–h) ref. 77 (copyright 2019, WILEY-VCH).

Similar to single component RTPs, many attempts have been reported to stabilize the triplet state through multiple interactions between two different structural units with complementary recognition parts. This section primarily summarizes the developments in the area of two-component RTP systems. An, Huang and coworkers reported that cocrystals formed by the assembly of melamine **ME** and **IPA** resulted in a stable framework *via* multiple interactions (Fig. 6).^91^ The two-component assembly **ME–IPA** exhibited URTP with *τ*_p_ of 1.91 s and *ϕ*_p_ of 24.3% under ambient conditions (Fig. 6c and d). The rigid framework confined the molecules in a three-dimensional network (Fig. 6e) and thus helped to limit *k*_nr_ of the triplet excitons and improved *k*_isc_. Similarly, the cocrystals of **ME–TPA** also presented excellent RTP features with *τ*_p_ of 1.09 s and *ϕ*_p_ of 19.4%. The RTP of the cocrystal is confirmed by the faster *k*_isc_ (9.3 × 10^7^ s^−1^) of the **ME–IPA** framework than that of the individual components **ME** (1.7 × 10^6^ s^−1^) and **IPA** (8.7 × 10^6^ s^−1^). It has to be noted that the SOC *ξ*(S_1_,T_*n*_) of **ME–TPA** and **ME–IPA** increased to 16.1 cm^−1^ and 33.9 cm^−1^, respectively, compared to the relatively lower values of monomers. If we compare **ME–IPA** and **ME–TPA**, the *k*_nr_ and *k*_p_ varied as 0.13 and 0.18 s^−1^, and 0.4 and 0.74 s^−1^, respectively. However, the co-assembly of **ME–PA** exhibited comparatively less RTP efficiency with *τ*_p_ of 0.68 s and *ϕ*_p_ of 0.82%. The advantages of the two-component phosphor enabled a simultaneous enhancement of *τ*_p_ and *ϕ*_p_.

A detailed mechanistic aspect of this significantly high RTP performance of the acid–amine cocrystals was explained by Yan and coworkers through TADF-assisted Förster resonance ET from the energy donor **ME** to the phosphor acceptor acids leading to a longer lifetime (Fig. 6f).^77^ Density functional theory calculations of the D–A assembly showed that the HOMO is located on **ME**, while the LUMO is on **PA**/**IPA**/**TPA** for **ME–PA**, **ME–IPA**, and **ME–TPA**, respectively. Furthermore, the possibility of ET was supported by the spectral overlap between the absorption of the acceptors (**PA**, **IPA**, and **TPA**) and the emission of **ME**. The emission peaks at 318, 373, and 342 nm have been shifted to 540, 524, and 554 nm in the delayed spectra with a lifetime of 0.43, 2.00, and 0.77 s, respectively, for **ME–PA**, **ME–IPA**, and **ME–TPA** (Fig. 6g and h). The enhanced RTP lifetime is strictly due to the TADF assisted efficient ET (76%) in the cocrystals. Interestingly, the cocrystals exhibited a strong afterglow lasting for many seconds (Fig. 6i). Two-component assembly promotes triplet state stabilization by additional charge-mediated hydrogen bonding and π–π stacking, resulting in enhanced RTP. A new strategy of multi-component URTP material utilizing both TADF and ET raises hope for further improvement.

The above section pointed out the recent developments in the area of organic crystalline phosphorescent materials. Many single- and multi-component assemblies have been examined to understand the underlying design principles for achieving high quantum yield and extended lifetime up to seconds. However, crystalline RTP materials lack processability and require tedious optimizations for practical applications. This situation demands alternative candidates to troubleshoot the existing barriers in such materials. Hence the organic materials chemists took up this challenge and introduced various new methods to improve RTP features, and the recent developments will be discussed in the following sections.^92^

### 3.2 Host–guest based organic phosphors

The effective control of the host and guest units to create a wide variety of soft materials drew much attention to supramolecular chemistry. Recently, researchers have successfully achieved RTP of pure organic host–guest systems by managing various intermolecular interactions.^93^ The host–guest interactions are highly selective because multiple factors such as size, shape, charge, polarity, *etc.* limit the host's inclusion. Therefore the selection of appropriate host and guest combinations is very critical. In general, the cavity of the host molecule specifically recognizes the guest molecule and provides a rigid environment to confine the guest molecules. The support is obtained not only from cavitands but other hosts such as small molecules and frameworks, also strongly supported phosphors. Organic host–guest based persistent RTP materials are mainly developed by minimizing *k*_nr_ of triplet excitons and keeping it smaller than the small *k*_p_.^93^ Since the fate of the triplet state is heavily dependent on nonradiative deactivation pathways of the guest and quenching by the diffusional motion of the host as well as molecular oxygen, the selection of a suitable host–guest combination remains challenging. Herein, the research progress of ORTP systems based on host–guest interactions is reviewed.

**Scheme 4:**
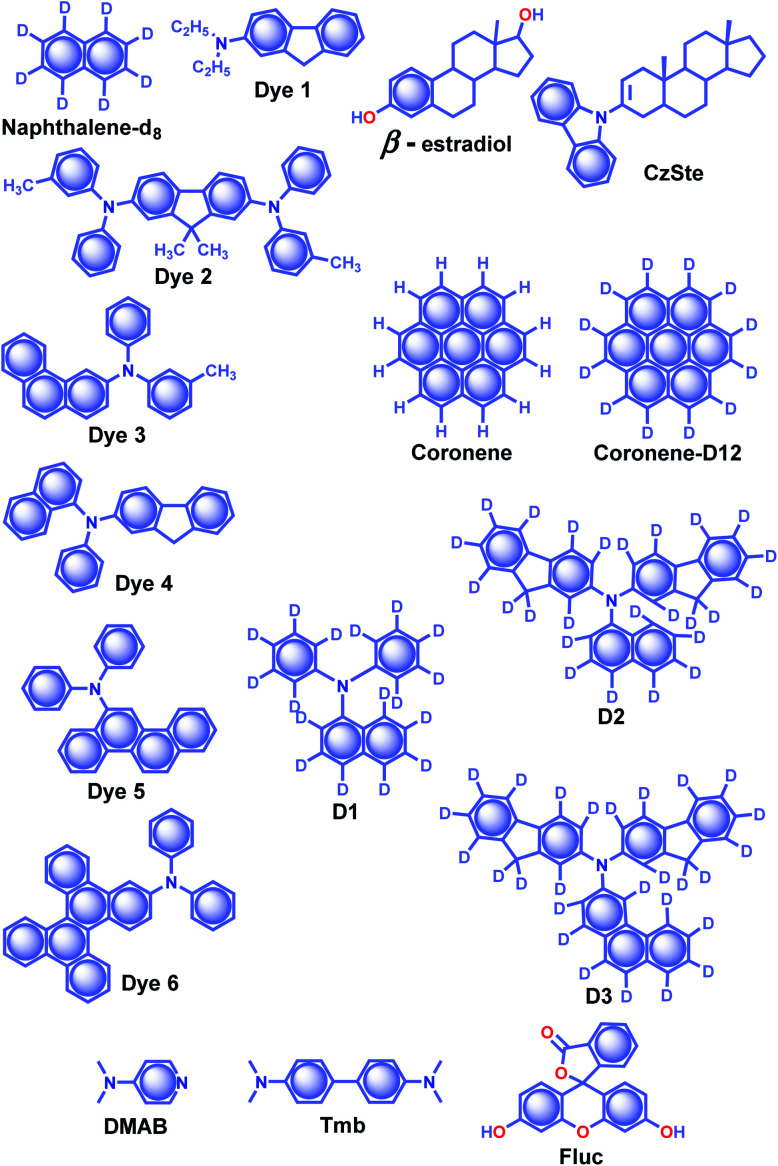
Chemical structure of β-estradiol, **CzSte** and **DMAB** (host) and naphthalene-d_8_, **Dyes1–6**, coronene, coronene-d_12_, **D1–3**, and **Fluc** (acceptor) molecules.

Alfimov and coworkers achieved long-lived RTP from arene-β-cyclodextrin (β-CD) cage–hydrocarbon complexes in the presence of oxygen.^94^ Among the different combinations, the complex naphthalene-d_8_-β-CD cage (Scheme 4) with various hydrocarbons showed variable RTP lifetime of 11.9 s for diadamantyl, 9.4 s for diamantine, and 10.3 s for adamantine in the presence of oxygen. However, the RTP lifetime of these complexes further increased in the absence of oxygen. The ternary complexes aggregate in water to form micro-particles, which prevent molecular motions and reduce the quenching effect from oxygen to achieve a longer lifetime. In another attempt, the same group demonstrated URTP from the supramolecular complex of naphthalene-d_8_-β-CD-cyclohexane (**I**) with a lifetime of around 16 s while the naphthalene-d_8_-β-CD-cyclohexane-benzophenone (**II**) complex showed 14.7 s.^95^ More interestingly, the long lifetime of complex **II** is attributed to the triplet–triplet (T–T) ET from benzophenone (donor) to naphthalene-d8 (acceptor).

**Fig. 7:**
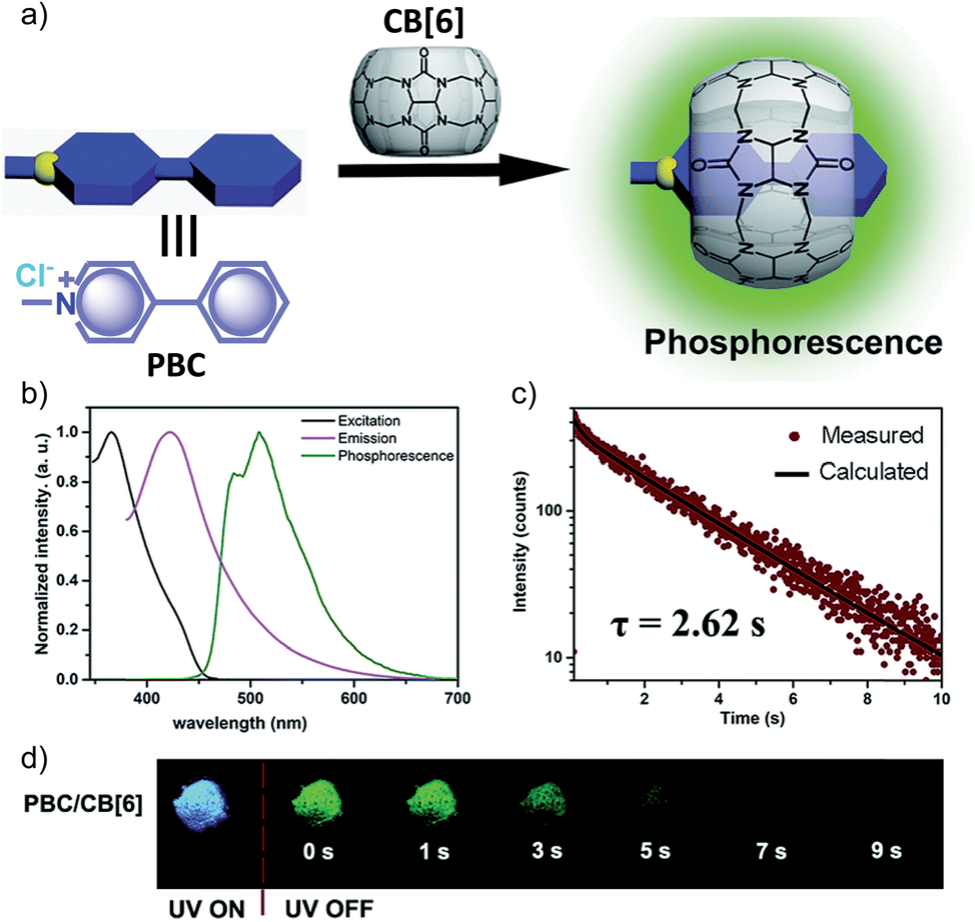
(a) Schematic illustration of the solid-state supramolecular strategy of **PBC** and **CB[6]** for URTP. (b) Excitation, emission, and phosphorescence spectra of **PBC**/**CB[6]** in the solid state. (c) Phosphorescence decay of **PBC**/**CB[6]** at 510 nm. (d) Photographs of **PBC**/**CB[6]** powder under 365 nm UV irradiation and at different time intervals after removal of the ultraviolet lamp. Reproduced with permission from ref. 96. Copyright 2019, Royal Society of Chemistry.

Another work in the field of host–guest supramolecular systems was reported by Liu and coworkers using cucurbit[6]uril (**CB[6]**) as the host and a heavy-atom-free phenylmethylpyridinium as the guest (Fig. 7a).^96^ The **PBC**/**CB[6]** complex formed by grinding showed a phosphorescence peak at 510 nm with an ultralong *τ*_p_ of 2.62 s and *ϕ*_p_ of 9.7% (Fig. 7b–d). The encapsulation in **CB[6]** promotes ISC in phenylmethylpyridinium, which in turn boosts the population of triplet excitons. Moreover, **CB[6]** provides a rigid matrix for the guest molecule to suppress molecular motions such as vibrations, rotations, and inter-collisions as well as to provide protection from oxygen. Eventually, the successful **PBC**/**CB[6]** complex prolonged the phosphorescence lifetime. Notably, the distinct lifetime and robust phosphorescence properties of **PBC/CB[6]** enabled the triple lifetime-encoding for information encryption and anti-counterfeiting applications.

The salient features of a steroidal compound, β-estradiol (Scheme 3), such as rigidity, oxygen barrier, high T_1_ energy, *etc.* motivated the research group of Adachi to use it as a host material to suppress the triplet quenching of phosphors.^97,98^ Besides, the use of a deuterated aromatic hydrocarbon as the guest minimized nonradiative deactivation. Red-green-blue persistent RTP with *τ*_p_ > 1 s, *ϕ*_p_ > 10% and a persistent RTP afterglow for several seconds was realized for dyes **1–6** (Scheme 4).^97^ In 2016, the same group introduced a new host molecule, 3-(*N*-carbazolyl)-androst-2-ene (**CzSte**) (Scheme 4), to enhance the performance of afterglow LEDs.^98^ To maximize the lifetime, the authors used coronene-d_12_ as the emitter and found a significant improvement in RTP features. The planar structure of coronene-d_12_ was found to be suitable for forming a rigid host matrix through intermolecular CH–π interactions with the steroid moiety of **CzSte**. The suppression of molecular vibration and nonradiative decay of the guest emitter resulted in an extended *τ*_p_ of 4.7 s and *ϕ*_p_ of 5.3%. Furthermore, the prepared host–guest system was found to be useful in LEDs yielding higher external quantum efficiency and longer afterglow.

Hirata and coworkers reported a new heavy atom-free organic molecular design consisting of a secondary amine as an RTP antenna substituted with different RTP centers **D1–3** (Scheme 4) having smaller T_1_ energy, exhibiting persistent RTP (Scheme 4).^99^ The notable feature of the molecular design is the steric hindrance introduced between the RTP antenna and the RTP center that decreases *k*_f_ and enables efficient *ϕ*_isc_. The authors cleverly extended the conjugation of the RTP antenna in anticipation of obtaining *k*_p_ > *k*_nr_. To validate the design strategy, RTP candidates (0.3 wt%) were dispersed in a β-estradiol host and a persistent emission was observed from 470 to 800 nm. The *ϕ*_p_ and *τ*_p_ of the host–guest systems varied as 11, 50, and 46%, and 1.60, 1.00, and 1.40 s for **D1**, **D2**, and **D3**, respectively.

Recently, a thermoresponsive RTP has been reported by taking advantage of ET and intermolecular CT between *N*,*N*-dimethylpyridin-4-amine **DMAP** (host) and *N*,*N*,*N*′,*N*′-tetramethylbenzidine **Tmb** (guest) (Scheme 4).^100^ The cocrystals of **DMAP** and **Tmb** with a mass ratio of 400 : 1 displayed blue RTP emission with *τ*_p_ and *ϕ*_p_ up to 2.1 s and 13.4%, respectively. In addition, the authors studied the concentration-dependent emission changes by incorporating an additional energy acceptor, fluorescein **Fluc** (Scheme 4), to form a ternary blend. Upon changing the concentration of **Fluc**, a colour-tunable afterglow from blue to yellow was realized. More interestingly, on heating, both **DMAP–Tmb** and **DMAP–Tmb–Fluc** exhibited turn-on RTP with increasing RTP lifetime from 1.4 s to 1.97 s. Here the enhanced intermolecular interactions in **DMAP–Tmb** and **DMAP–Tmb–Fluc** played a significant role in enhancing the phosphorescence lifetime. Furthermore, the thermoresponsive nature of the host–guest RTP materials has been used for multi-colour thermal printing.

Metal–organic frameworks (MOFs) are capable of encapsulating guest species in their cavities, and the guest confinement can deliver a significant improvement in RTP. Along these lines, Kabe, Adachi, and coworkers demonstrated a long-lived emission from triplet excitons achieved by encapsulating coronene in a zeolitic imidazolate framework (ZIF-8) host.^101^ It is confirmed that the coronene wholly isolated within the pores of the MOF suppresses the nonradiative decay and molecular vibrations, enabling long-lived RTP. Coronene@ZIF-8 exhibited *τ*_p_ of 7.4 s, while an extended lifetime of 22.4 s was achieved for coronene-d_12_@ZIF-8 under the same experimental conditions. The vibrational energy of the C–D stretching mode is lower than that of the C–H stretching mode, and hence it helps coronene-d_12_@ZIF-8 to show enhanced lifetime. Moreover, the temperature-dependent lifetime measurement confirmed the suppression of nonradiative deactivation of coronene.

**Fig. 8:**
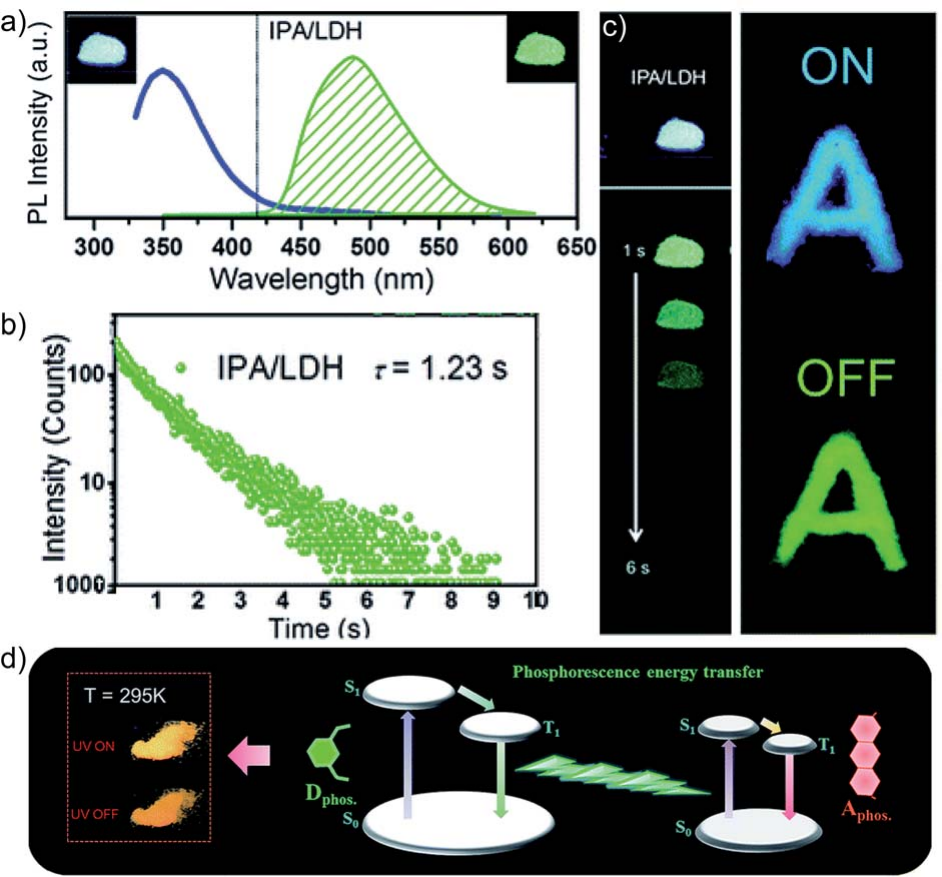
(a) Steady-state emission (left) and phosphorescence (right) spectra of **IPA/**LDH nanohybrids (insets show the corresponding photographs). (b) Phosphorescence decay profile of **IPA/**LDH (*λ*_mon_ = 489 nm, *λ*_ex_ = 320 nm). (c) The letter ‘A’ made with **IPA/**LDH can be unmistakably identified by the naked eye after the excitation is switched off. (d) Schematic representation of the proposed mechanism for PET. Reproduced with permission from ref. 102. Copyright 2017, Royal Society of Chemistry.

In 2017, Yan and coworkers explored a phosphorescence ET by incorporating donor and acceptor guest molecules in the interlayer nanogallery of an inorganic graphene-like layered double hydroxide (LDH) host material (Fig. 8).^102^ The authors used different benzene dicarboxylic acid isomers, namely **IPA** (Scheme 2), **TPA**, and **PA** (Fig. 6a), as potential donors assembled into the interlayer of the Zn–Al-LDH host by the co-precipitation method. Interestingly, among the nanohybrids, the **IPA**/LDH showed a green phosphorescence emission with the longest *τ*_p_ up to 1.2 s and *ϕ*_p_ of 3.02% (Fig. 8b–d). An H-type aggregation between **IPA** dimers and LDH nanosheets stabilized the lowest triplet excited state and minimized the nonradiative decay to prolong the RTP lifetime. Besides, **IPA**/LDH showed thermoresponsive RTP upon varying the temperature from 295 to 335 K. Subsequently, the co-intercalation of eosin Y as an energy acceptor with the **IPA** energy donor into the nanogalleries of LDH nanosheets imparted excellent triplet–triplet ET (*E*_P_ = 99.7%) (Fig. 8e).

**Scheme 5:**
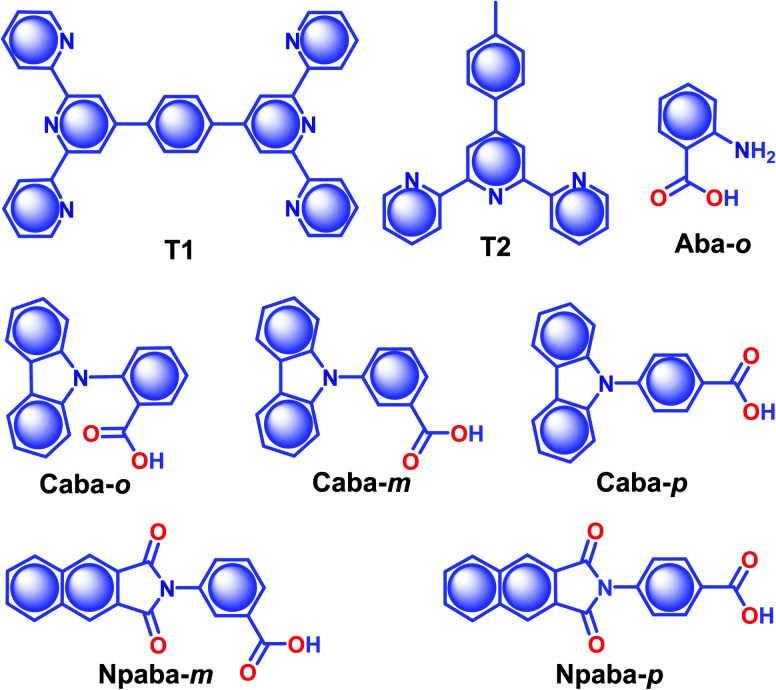
Chemical structure of **T1**, **T2**, **Aba-o**, **Caba-o**, **Caba-m**, **Caba-p**, **Npaba-m**, and **Npaba-p**.

Co-assembly of RTP inactive terpyridine-derivatives **T1** and **T2** (Scheme 5) with LAPONITE® (LP) nanoclay through solvent-free mechanical grinding significantly enhanced the RTP to exhibit green (**T1**@LP) and blue-green (**T2**@LP) emissions.^103^ The long RTP lifetime decay component of both the hybrid materials was around 1.1 s with a strong afterglow lasting for more than 10 seconds. The encapsulation of the emitters in LP helped to achieve *ϕ*_p_ of 2.96 and 1.86% for **T1**@LP and **T2**@LP, respectively. A transformation from *trans*–*trans* to *cis*–*trans* configuration of **T1** upon protonation has led to a marginal increase in spatial separation of the HOMO and LUMO and eventually narrowed down Δ*E*_ST_ to facilitate ISC. Moreover, the hydrogen bonding between **T1** and LP reduces the nonradiative decay and protects the triplet excitons. Zhang and coworkers came up with a hybrid material by encapsulating various RTPs (**Aba-o**, **Npaba-m**, **Npaba-p**, **Caba-o**, **Caba-m**, and **Caba-p**) in [Al(DMSO)_6_]X_3_, where X is Cl^−^ or Br^−^ (Scheme 5).^104^ The new approach resulted in a very high RTP lifetime and luminescence quantum yield. The heavy atom effect has a profound impact in this series: as compared to Cl^−^ hybrids, the analogues with Br^−^ yield high *ϕ*_p_ and a shortened *τ*_p_. The values of *τ*_p_ and *ϕ*_p_ varied for **Aba-o**/Cl (1.1 s, 2.3%), **Npaba-m**/Cl (1.12 s, 3.9%), **Npaba-p**/Cl (1.26 s, 4.8%), **Caba-o**/Cl (1.81 s, 4.9%), **Caba-m**/Cl (1.84 s, 8.4%) and **Caba-p**/Cl (1.89 s, 2.5%). The presence of many different types of weak interaction between the matrix and RTPs greatly supports RTP by suppressing nonradiative decay.

### 3.3 Polymer-based organic phosphors

The crystalline URTP materials have drawbacks in terms of reproducibility, processability, and flexibility, which significantly impedes the development of crystalline URTP materials for practical applications.^105^ To overcome these fundamental barriers, special attention has been paid to the development of organic polymeric materials capable of URTP. Recently, significant breakthroughs have been achieved by prolonging the lifetime of polymeric materials through homopolymerization, ring-opening polymerization, covalent cross-linking reaction, and radical binary copolymerization, as well as embedding small molecules into a rigid polymer matrix.^105^ Polymer-based RTP materials have been receiving increased attention because of the large molecular weight of the polymer and the availability of functional group chains that can help to rigidify the molecular vibrational and rotational motions of the phosphors. Moreover, it can reduce the quenching effects of oxygen and moisture from the ambient environment, allowing the triplet excitons to survive long enough to achieve prolonged lifetimes. More importantly, polymer-based RTPs exhibit easy processability, excellent flexibility, and high thermal stability as well.

**Scheme 6:**
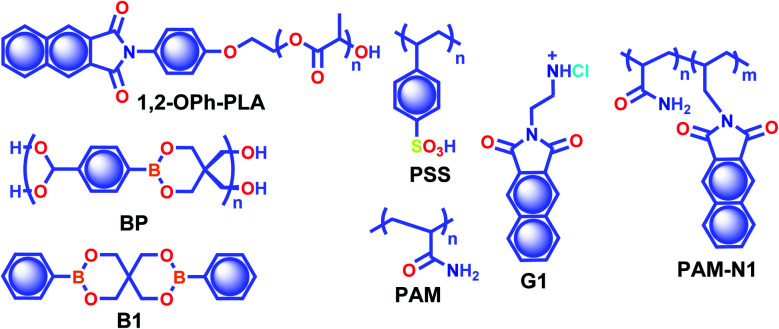
Chemical structure of polymers **1,2-OPh-PLA**, **BP**, **PSS**, **PAM**, **G1**, and **PAM-N1**, and model derivative **B1**.

In 2016, Chen *et al.* applied the intramolecular CT (ICT) state of *N*-substituted naphthalimide polylactic acid-based polymers to obtain RTP enhancement.^40^ It has been noticed that either the ICT state or a heavy atom (Cl or Br) can ensure enhanced ISC and thus strong RTP. In this series, polymer **1,2-OPh-OLA** (Scheme 6) showed strong ICT and a favourable Δ*E*_ST_ to support RTP. The existence of an ICT state acted as a bridge between the excited singlet and triplet states and thus accelerated ISC leading to *τ*_p_ of 1.12 s. Additionally, *N*-substituted naphthalimides conjugated with natural biomacromolecules such as chitosan and bovine serum albumin also displayed RTP and found useful application in time-resolved bioimaging.

Ogoshi and coworkers reported URTP with a lifetime up to 1.22 s for poly(styrene sulfonic acid) **PSS** in the dry solid-state (Scheme 6).^106^ The observed lifetime is one of the most extended RTP lifetimes for non-doped ORTP polymers. The sulfonic acid group in the polymer can form strong inter/intrachain hydrogen bonds in the solid-state that reduce the nonradiative decay and eventually lead to ultralong RTP. The RTP lifetime depends on the introduction ratio of sulfonic acid groups. As the ratio is increased, the phosphorescence lifetime became longer due to strong hydrogen bond formation between sulfonic acid groups. Furthermore, the reversible RTP *via* uptake and removal of water contributed to the lifetime-encoding application. Detailed studies revealed that deuteration of SO_3_H and exchanging SO_3_Na or SO_3_K for SO_3_H resulted in an increased RTP lifetime. In contrast, a decrease in RTP lifetime was noticed when **PSS** was neutralized with NaOH or KOH.

Cai *et al.* demonstrated that the ionic cross-linking between chromophores is critically supportive in suppressing nonradiative transitions for URTP, and by utilizing the concept, a lifetime of 2.1 s for an amorphous polymer is obtained.^107^ The replacement of **PSS** with different ions such as Li^+^, K^+^, Rb^+^, NH^4+^, Mg^2+^, Ca^2+^, Al^2+^, and Gd^2+^ imparted a significant effect on the URTP of polymers. The size of the ionic radius is found to control the RTP features, and as the size increases, the URTP lifetime also gradually decreases. The replacement with Li^+^ and Mg^2+^ resulted in a lifetime of 1.3 and 1.1 s, respectively, and thereafter a gradual decrease in the lifetime is observed. It has been concluded that even though the large ionic radius prevents the prolonged URTP, the high ion charge state is found to be supportive. Hence a balance between the ionic radius and charge state can significantly alter the lifetime values. Here the *k*_nr_ is at least one order of magnitude higher than *k*_p_, indicating that the former one played a dominant role in manipulating the URTP of ionic polymer phosphors. The results of ionic cross-linking assisted URTP have even been extended to nonaromatic ionic polymers and it was found that **PAANa** (Scheme 6) with blue URTP has *τ*_p_ of 1.4 ms and 2.1 s, respectively, when monitored at 450 nm and 480 nm bands.

Recently, boronic acid/ester-based organic phosphors also excelled as strong RTP candidates. Along these lines, Kubo and coworkers reported boronate particles **BP** (Scheme 6) as a self-assembled URTP system in both solid state and dispersion in water.^108^ Solid **BP** showed phosphorescence peaks located between 450 and 550 nm with a long-lived *τ*_p_ of 1.95 s and *ϕ*_p_ of 5% under ambient conditions. The RTP properties of **BP** were compared with a model derivative, 3,9-dibenzo-2,4,8,10-tetraoxa-3,9-diboraspiro[5.5]undecane **B1** (Scheme 6). Theoretical calculation and the crystal structure of **B1** suggest that boron-containing CT interactions and the presence of intermolecular electron coupling facilitate RTP. Notably, grafting rhodamine B fluorophores on the surface enabled the ET process from the triplet excited state of **BP** to the singlet state of the fluorophore resulting in an afterglow composed of dual luminescence at ∼500 and 600 nm.

In 2020, Ling and coworkers reported a colourful afterglow through regulation of clusterization-triggered RTP of non-conjugated amorphous polyacrylamide (**PAM**).^109^ The emission features of these non-conjugated polymers containing carbonyl and amine groups depend on the aggregation, which can result in electronic interactions by n–π and π–π interactions. Furthermore, the clusterization of amides can form a rigid conformation of polymer chains, which is helpful to inhibit nonradiative decay of excitons and to stabilize the excited state through hydrogen bonding. When PAM was blended with naphthalimide **G1** (Scheme 6), URTP with *τ*_p_ up to 1.7 s and *ϕ*_p_ of 13.4% was observed in solid powders. Computational studies revealed the possibility of a clusterization-triggered phosphorescence mechanism. When naphthalimide was covalently linked with the PAMs, **PAM-N1** (Scheme 6) exhibited a visible-light-excited URTP with *τ*_p_ of 1.5 s and *ϕ*_p_ of 12%.

**Fig. 9:**
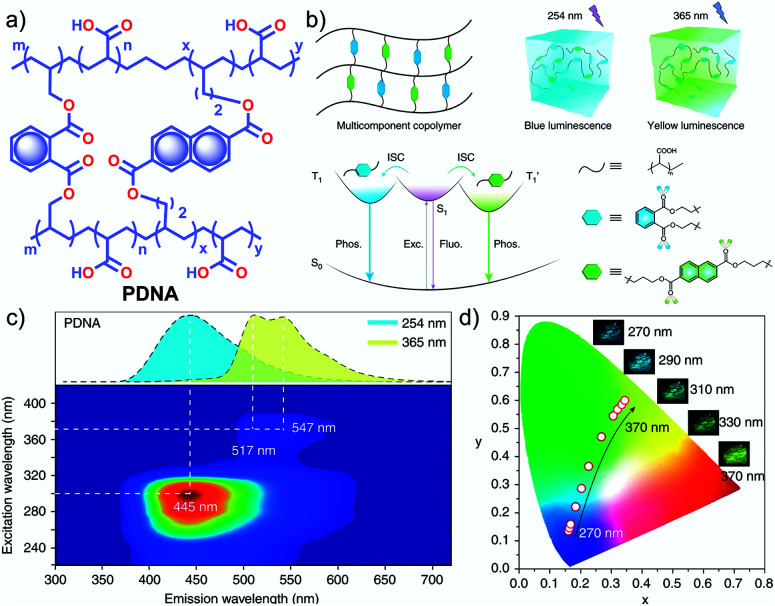
(a) Chemical structure of **PDNA**. (b) Schematic illustration of the proposed mechanism of colour-tunable URTP of **PDNA**. (c) Excitation-phosphorescence mapping of **PDNA** under ambient conditions; the inset displays the phosphorescence spectra excited at 254 nm (blue) and 365 nm (yellow). (d) CIE chromaticity diagram for **PDNA** with excitation varied from 270 to 370 nm; the inset shows the UOP photographs of **PDNA** excited at various wavelengths. Reproduced with permission from ref. 110. Copyright 2020, Springer Nature Limited.

In 2020, Gu *et al.* discovered a colour-tunable URTP in polymers through multi-component cross-linked polymerization by using acrylic acid and multiple luminophores.^110^ A copolymer, **PDNA** (Fig. 9a), prepared using vinyl derivatives of naphthalene (MND) and benzene (MDP), and acrylic acid (MND/MDP/AA ratio 1/200/10 000) displayed an excitation dependent multi-colour RTP emission spanning from blue to yellow with a long-lived *τ*_p_ of 1.1 s and *ϕ*_p_ of 23.2% (Fig. 9b–d). As the ratio of MND/MDP/AA varied, the phosphorescence intensity gradually decreased. Two other polymers with varying ratios of MND/MDP/AA, namely **PDNA-5** (1/5/1000) and **PDNA-10** (1/10/1000), also exhibited excellent RTP features with *τ*_p_ of 1.22 and 1.07 s and *ϕ*_p_ of 13 and 37.5%, respectively. The excitation spectra of **PDNA** revealed that the blue and yellow emission bands originate from two entirely different excited triplet states of benzene and naphthalene components in the polymer and this is confirmed by the detailed analysis of the individual polymers. The numerous carbonyl and hydroxyl groups of **PDNA** assist in forming inter- or intramolecular hydrogen bonds with polyacrylic acid chains. Thus the eventually created rigid environment suppressed the nonradiative decay of the excited state and prevented the quenching of triplet excitons. The hydrogen bonding assisted RTP in **PDNA** was revealed by a significant decrease in RTP features in the presence of moisture, which breaks the hydrogen bonds between the polymer chains. The overall tunable emission URTP achieved by **PDNA** is demonstrated in Fig. 9d.

### 3.4 Polymer supported organic phosphors

**Fig. 10:**
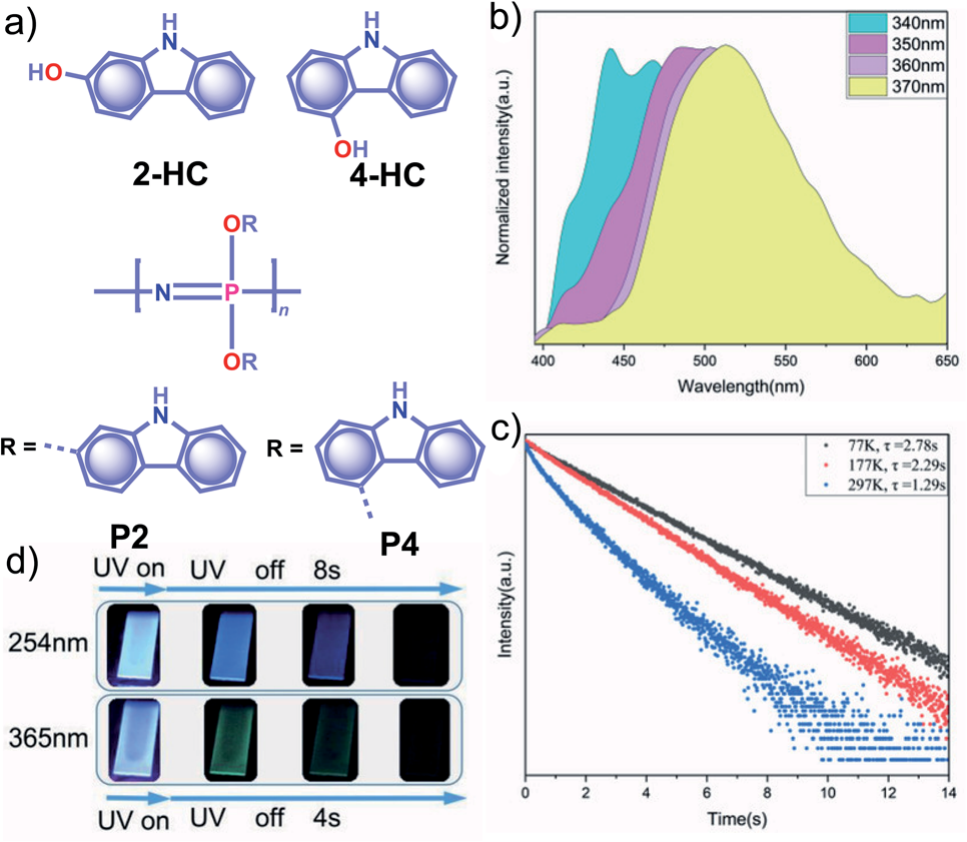
(a) Chemical structure of monomers **2-HC** and **4-HC**, and polymers **P2** and **P4**. (b) Excitation wavelength-dependent phosphorescence spectra and (c) temperature-dependent phosphorescence decay curves of **PVA-100-P4-1** film. (d) Photographs of persistent luminescence of **PVA-100-P4-1** film under ambient conditions. Reproduced with permission from ref. 112. Copyright 2020, WILEY-VCH.

Apart from incorporating the functional phosphors as a part of the polymer backbones, polymer-supported phosphors also found URTP active. In 2019, Zhao and coworkers achieved URTP from **2-HC** (Fig. 10a) by coassembling with polyvinyl alcohol (**PVA**).^111^ The confinement of **2-HC** in **PVA** restricts the molecular motions to stabilize the triplet state and thereby generate URTP (*τ*_p_ = 1.21 s and *ϕ*_p_ = 16%) with an afterglow lasting for more than four seconds in the dark. The same group demonstrated the excitation-dependent persistent emission by constructing multiple emission centers in polymeric systems with hydrogen bonding.^112^ A polyphosphazene derivative, **P4**, containing carbazole unit **4-HC** was synthesized and mixed with **PVA** to develop a composite, **PVA-100-P4-1** (Fig. 10a). As shown in Fig. 10b, an excitation wavelength-dependent (340 to 370 nm) redshift of phosphorescence (468 to 522 nm) was observed for **PVA-100-P4-1**. The afterglow of **PVA-100-P4-1** persisted for 12 s, and the corresponding *τ*_p_ reached 1.29 s with *ϕ*_p_ of 1.0% (Fig. 10c). The presence of strong hydrogen bonding between the polyphosphazene polymer chains and PVA plays a critical role in the afterglow. The *k*_nr_ for the **PVA-100-P4-1** film has significantly come down to 0.77 s^−1^ as compared with that of the **PVA-100-4HC-1** precursor film (2.59 s^−1^). An excitation wavelength-dependent persistent luminescence colour from blue to green indicates the presence of multiple radiation channels in the system (Fig. 10d). Even though the monomer **2-HC–PVA** composite exhibited a longer lifetime, the corresponding polymer **PVA-100-P2-1** failed to extend the lifetime.

**Fig. 11:**
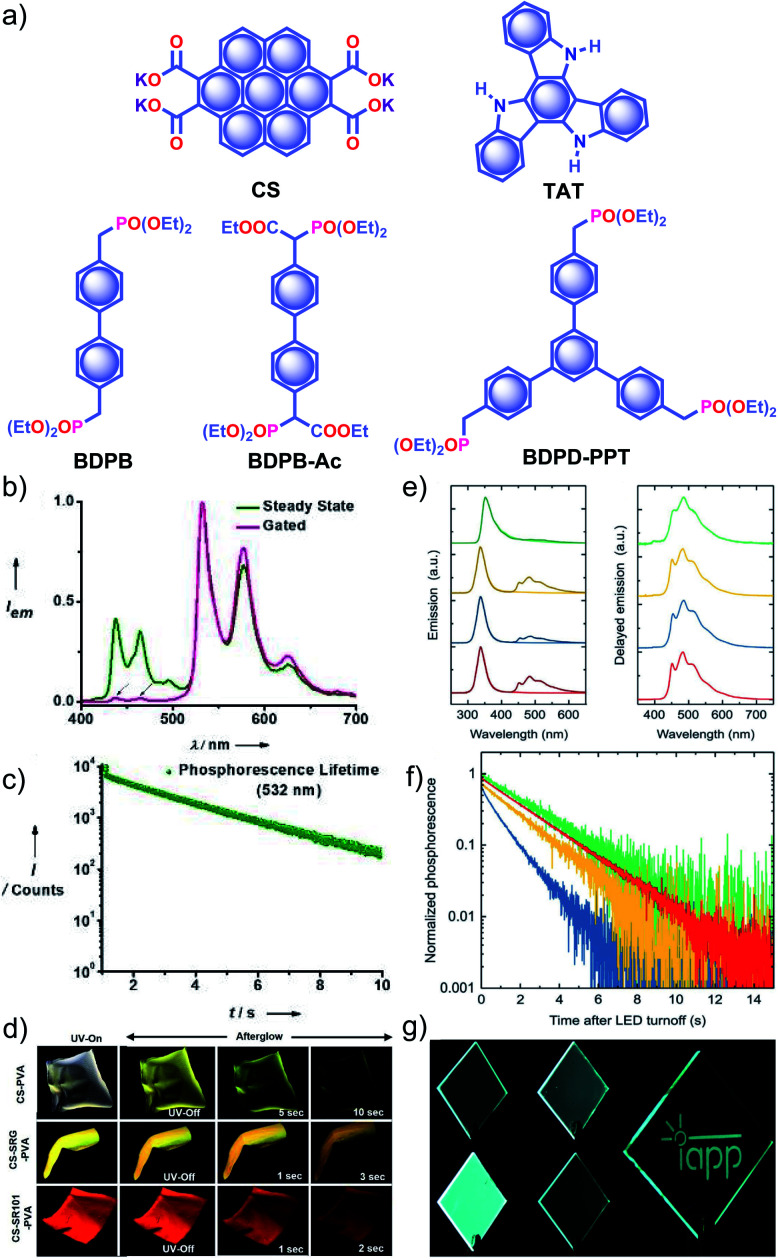
(a) Chemical structure of **CS**, **TAT**, and aromatic phosphonates **BDPB**, **BDPB-Ac**, and **BDPD-PPT**. (b) Steady-state and gated emission spectra and (c) phosphorescence decay profile of **CS–PVA** film. (d) Photographs of **SRG/SR101** doped **CS–PVA** hybrid films showing ambient afterglow properties. (e) Emission spectra of aromatic phosphonates under an aerated (light colour) and a nitrogen atmosphere (dark colour) (left), and delayed spectra showing RTP in an Exceval, aerated atmosphere (right) along with the corresponding (f) phosphorescence decays. (g) Photographs of **BDPB-Ac**, **BDPB**, and **BDPD-PPT** in Exceval, and delayed phosphorescent image written by masked UV illumination in a PMMA:**BDPB** sample covered with Exceval. Reproduced with permission from (a–d) ref. 113 and (e–g) ref. 115. Copyright 2020, WILEY-VCH.

Recently, George and coworkers reported a delayed sensitization assisted triplet to singlet ET in polymer-supported D–A pairs.^113^ The authors used a **PVA** matrix to host coronene tetracarboxylate salt **CS** as a triplet energy donor and fluorescent dyes sulpharhodamine101 **SR101** and sulpharhodamine G **SRG** as acceptors to demonstrate PET (Fig. 11a). Since both the donor and acceptors are water-soluble dyes having polar side-groups, it facilitates co-assembly with **PVA***via* ion-dipole and hydrogen bonding interactions. The **CS–PVA** hybrid showed a phosphorescence band in the range of 500 to 700 nm with an average ultralong *τ*_p_ of 2.46 s with *ϕ*_p_ of 23.4% (Fig. 11b and c). The emission spectra of **SR101**/**SRG** doped **CS–PVA** films showed a gradual decrease of **CS** phosphorescence emission with an enhancement of acceptor emission in the 550–700 nm region due to ET from the triplet state of a donor to the singlet state of the acceptors. The hybrid thin films are self-standing and flexible with stable afterglow features (Fig. 11d). The same group reported deep blue URTP from triazatruxene **TAT** (Fig. 11a) with an average *τ*_p_ of 2.26 s and *ϕ*_p_ of 17.5% in a **PVA** matrix.^114^ The deep-blue emission of **TAT–PVA** hybrid films persisted over 10 s, pointing to the RTP from the spatially isolated **TAT** in the **PVA** matrix, supported by strong hydrogen-bonding interaction between **TAT** and **PVA**. Interestingly, a mixed RTP hybrid of **CS–TAT–PVA** exhibited excitation-dependent multi-colour afterglow emission, including an ambient white afterglow with the CIE coordinates (0.29, 0.33).

With the understanding of ORTP of amorphous polymer materials, Reineke and coworkers reported a new family of halogen-free organic luminescent derivatives called aromatic phosphonates (Fig. 11a).^115^ A series of aromatic phosphonates 4,4′-bis(diethylphosphonomethyl)biphenyl **BDPB** and its derivatives **BDPB-Ac** and **BDPD-PPT** were embedded in a polymethyl methacrylate (PMMA) host matrix and covered with Exceval to prepare a hybrid RTP system (Fig. 11e–g). The presence of PMMA and Exceval ensures hydrogen bonding to rigidify the matrix, acts as an oxygen barrier layer, and efficiently suppresses vibrational dissipation to achieve bright long-lived RTP. In the PMMA matrix, when excited at 300 nm, *τ*_p_ varied as 1.7 s (**BDPB-Ac**), 1.8 s (**BDPB**), and 2.1 s (**BDPD-PPT**) and further varied as 2.0 s, 2.4 s, and 2.6 s, respectively, when excited at 275 nm (Fig. 11f). Furthermore, RTP of the aromatic phosphonates revealed that the main reason for the long lifetimes is the diethyl-phosphonomethyl units. Interestingly, the lifetime is increased by around 250 ms upon an increase from two to three phosphonate groups.

## 4. Comparative study

**Fig. 12:**
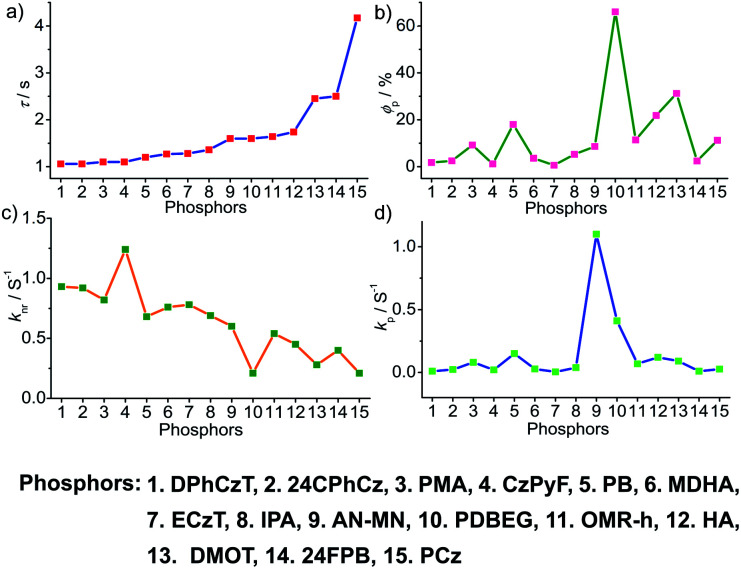
Analysis of (a) *τ*_p_, (b) *ϕ*_p_, (c) *k*_nr_ and (d) *k*_p_ of single-component organic phosphors.

To obtain a deeper understanding of the decay pathways, their trends, and their effect on *τ*_p_ and *ϕ*_p_, single-component organic phosphors have been selected for detailed analysis (Fig. 12). However, a few cases with incomplete data are omitted for clarity of the discussion. A comparison made by arranging in the ascending order of lifetimes showed that a variation in *τ*_p_ is mostly reflected in *k*_nr_ than *k*_p_, and this might be due to the significant contribution by *k*_nr_ in this particular class of molecules (Fig. 12a and c). A detailed analysis indicates that there exists a correlation between*τ*_p_ and *k*_nr_, as well as *ϕ*_p_ and *k*_p_. Compared with the increasing value of *τ*_p_, *k*_p_ irregularly varied between 0.01 and 0.15 s^−1^, while *k*_nr_ varied inversely as 1.24 to 0.21 s^−1^, with a few exceptions, in this series. This magnitude difference of *k*_nr_ and *k*_p_ imparts an upper hand for *k*_nr_ over *k*_p_ on lifetime values (Fig. 12c and d). Even though for single component phosphors *k*_nr_ > *k*_p_, the value of *ϕ*_p_ varied almost in line with *k*_p_ (Fig. 12b and d). We consider this discussion as a qualitative one due to the inconsistencies in sample preparation, quality of crystals, measuring conditions, reproducibility, and so on. Hence, a detailed study is highly required to reveal the underlying details of RTP to go further.

**Fig. 13:**
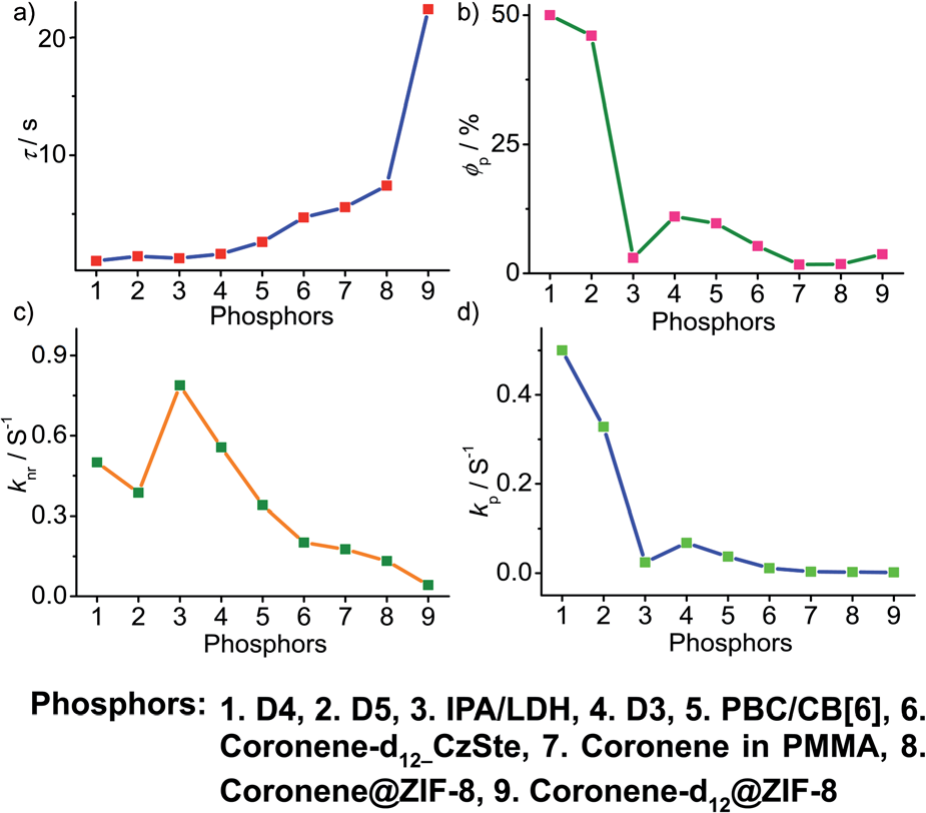
Analysis of (a) *τ*_p_, (b) *ϕ*_p_, (c) *k*_nr_ and (d) *k*_p_ of host–guest based phosphors.

A comparison of the decay pathways connected with *τ*_p_ and *ϕ*_p_ showed that the variations in decay parameters of the host–guest systems are in line with the expected trend (Fig. 13). The analysis indicates that except for a few cases, the variation of lifetime is reflected on both *k*_nr_ and *k*_p_ (Fig. 13a, c and d). In this series, the value of *k*_nr_ varies between 0.79 and 0.04 s^−1^, while *k*_p_ gradually decreased from 0.5 to 0.002 s^−1^ (Fig. 13c and d). However, a longer lifetime is achieved by lowering both *k*_nr_ and *k*_p_. The presence of the hosts is found supportive to enhance RTP lifetimes. Moreover, the values of both *k*_nr_ and *k*_p_ are observed to be almost in the same range. It has to be noticed that irrespective of the host–guest systems considered, a clear trend is reflected in the value of *k*_p_ and *ϕ*_p_ (Fig. 13b and d). Compared to single-component organic phosphors, the magnitude difference between *k*_nr_ and *k*_p_ is narrow, and therefore, we can assume that the suppression of *k*_nr_ is efficient in the host–guest based RTP systems due to the rigidification of the confined phosphors.

**Fig. 14:**
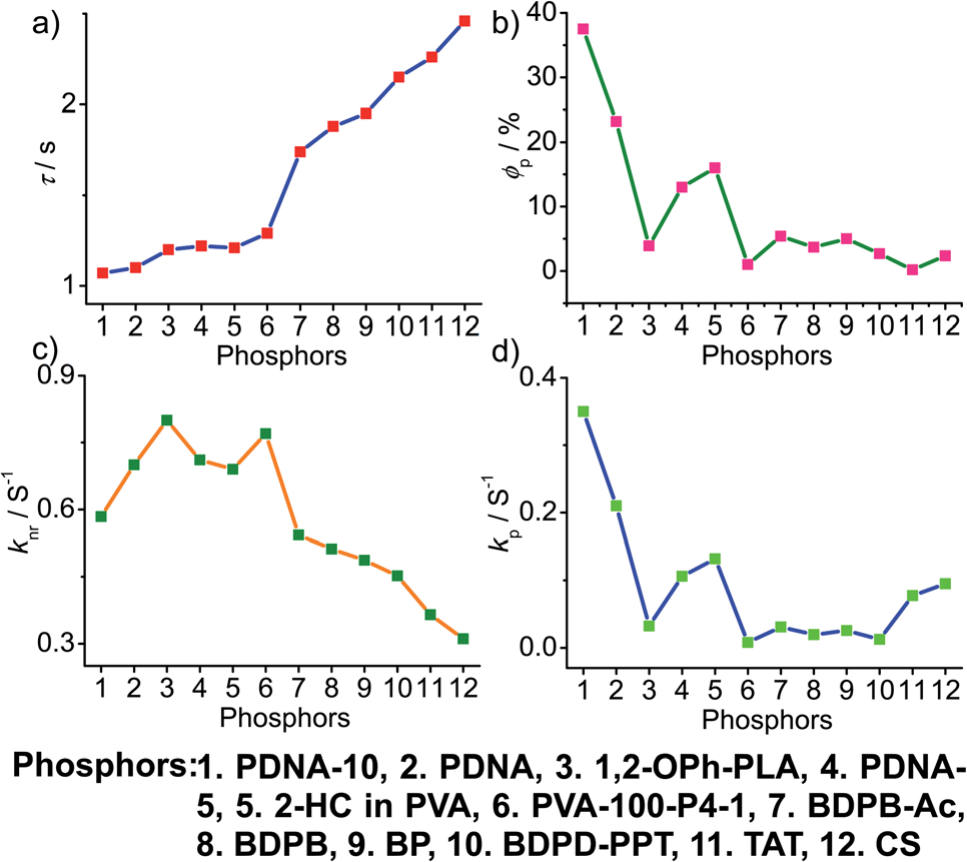
Analysis of (a) *τ*_p_, (b) *ϕ*_p_, (c) *k*_nr_ and (d) *k*_p_ of polymer and polymer-supported phosphors.

In the case of polymer related systems, a reasonable correlation between *τ*_p_ and *ϕ*_p_ is visible (Fig. 14a and b). As the *τ*_p_ is increased from 1 to 2.46 s, an almost steady decrease in *ϕ*_p_ from 37.5 to 2.34% is noticed (Fig. 14a and b). Similar to host–guest systems, the comparison of the decay parameters with *τ*_p_ and *ϕ*_p_ indicates a direct link between *τ*_p_ and decay rates *k*_nr_ and *k*_p_ (Fig. 14). The variation of the lifetime is reflected in the values of both *k*_nr_ and *k*_p_, which decreased from 0.8 to 0.3 s^−1^ and 0.35 to 0.01 s^−1^, respectively (Fig. 14a, c and d). In polymer and polymer-supported systems, the distinct difference in the magnitude of *k*_nr_ and *k*_p_ is visible. Even though *k*_nr_ > *k*_p_, long lifetimes for polymer-based phosphors have been achieved by lowering the values of both *k*_nr_ and *k*_p_. Interestingly, a similar trend noticed for *ϕ*_p_ and *k*_p_ points to the dependence of *ϕ*_p_ more on *k*_p_ than *k*_nr_ (Fig. 14b and d). The comparison of three different classes of organic phosphors, crystalline assemblies of single molecules, host–guest, and polymer-based systems revealed that compared to other phosphors, hosts provide a strong support to the guest phosphors and reduce *k*_nr_ as close to *k*_p_ as possible. It indicates the need for newer designs to minimize *k*_nr_ values to improve the efficiency of organic phosphors. A meaningful conclusion on the various parameters influencing both *ϕ*_p_ and *τ*_p_ can be generated only by comparing the SOC and *k*_isc_ values of the phosphors along with *k*_p_ and *k*_nr_. However, the lack of data in the literature reports prevented us from having such a detailed discussion.

## 5. Applications

**Scheme 7:**
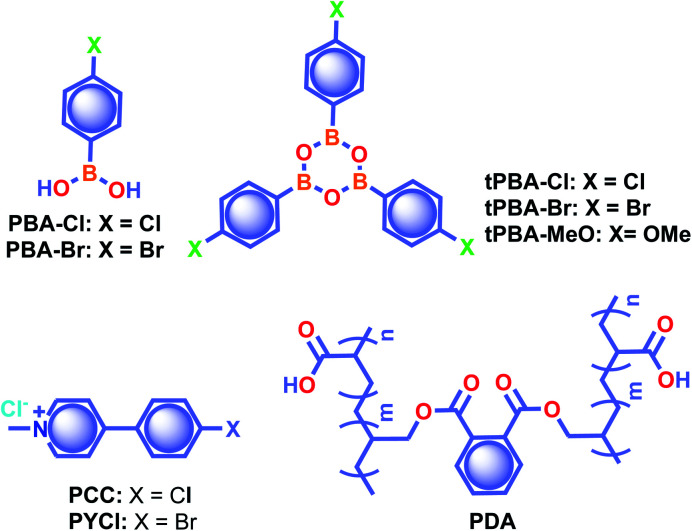
Chemical structure of the supporting phosphors used for various applications.

**Fig. 15:**
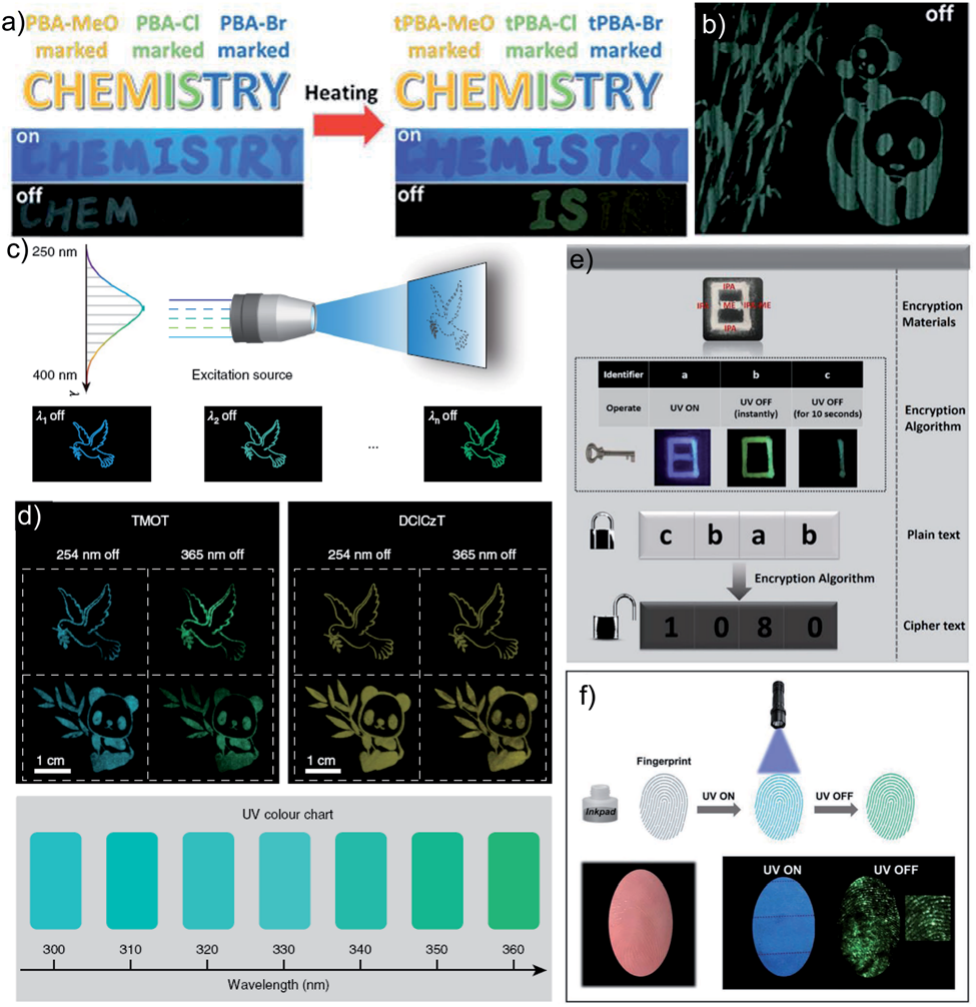
(a) Demonstration of security documents with **tPBA-MeO**, **tPBA-Cl** and **tPBA-Br** before and after heating. (b) A pattern of panda printed with **PBA-MeO** for 8 cycles after stopping excitation. (c) The UOP photographs of peace dove and panda patterns, recorded with the **TMOT** (left) and **DClCzT** (right) as the ink. (d) UV colour chart showing the ability of TMOT crystalline powder to visually detect specific wavelengths in the UV region. The schematic diagram of (e) encryption to decryption and (f) back-free fingerprint identification and luminescent images of the fingerprint with a special inkpad under UV excitation and after stopping UV excitation using **IPA**, **MA** and **IPA-MA**. Reproduced with permission from (a and b) ref. 41 (copyright 2017, Royal Society of Chemistry), (c and d) ref. 87 (copyright 2019, Springer Nature Limited) and (e and f) ref. 77 (copyright 2019, WILEY-VCH).

As mentioned in the introduction, the sudden developments of URTPs in recent years have engaged them in various potential applications, including organic electronics, optical recording, anti-counterfeiting, bioimaging, and sensing.^116–121^ Since many reviews have already summarized the applications of various ORTPs,^21–25,59,80–85^ we discuss only the recent developments of very efficient ORTPs. Li and coworkers utilized the strong interactions of aryl boronic acids *via* hydrogen bonds to develop an inkjet printing technology suitable for optoelectronic displays.^41^ The comparable intensities of the RTP crystals and samples from solvent evaporation enabled the use of the aryl boronic acid-derived phosphors as a low-cost ink. The fluorescence colour and intensity difference associated with the thermally formed boroxines **tPBA-MeO**, **tPBA-Cl**, and **tPBA-Br** (Scheme 7) from different monomer phosphors **PBA-MeO** (Scheme 2), **PBA-Cl**, and **PBA-Br** (Scheme 7) helped to make distinguishable RTP patterns (Fig. 15a). As a significant advantage, the brightness of the inkjet-printed designs can be improved by cyclic printing (Fig. 15b). The scalable synthesis of phosphors, stability, accuracy, and reproducibility of the images point to an impressive printing process. Besides, **PBA-MeO** exhibited less toxicity when fed to *Bombyxmori* silkworms, making it a potential candidate for biological applications. The excitation-dependent UOP feature of the phosphors has been used for multicolor display applications.^87^ By using **TMOT** and **DClCzT** powders as solid ink for silk-screen printing, different patterns, including a peace dove, panda, Cp rings, and butterfly, were fabricated (Fig. 15c). Since **TMOT** is excitation-dependent UOP active, a change in the excitation wavelength from 254 to 365 nm resulted in a drastic emission color difference (Fig. 15d). The change in the phosphorescence color from sky-blue to green demonstrates as a tool for the detection of UV light.

**Fig. 16:**
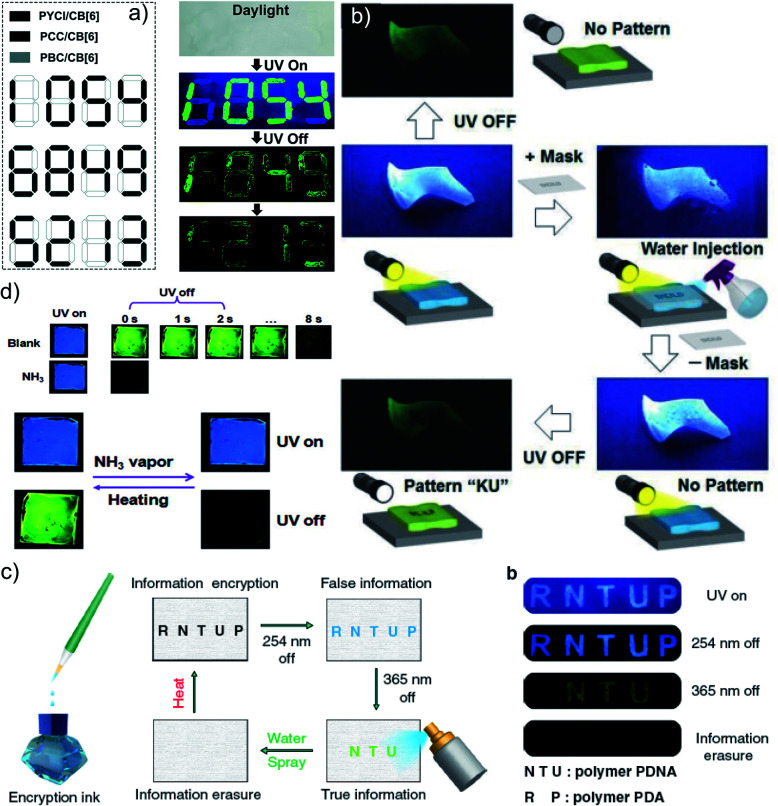
Lifetime-encoding for security applications using (a) **PYCl/CB[6]**, **PCC/CB[6]** and **PBC**/**CB[6]**, and (b) **PSS**. (c) Process of information encryption by using the multi-component copolymer **PDNA** (NTU) and **PDA** (RP) as encryption ink and long-lived luminescence photographs of letters (RNTUP and NTU) before and after switching off the UV light of 254 and 365 nm. (d) Photographs of **PAM-N1** films in NH_3_ vapors, taken under 365 nm UV light, and after turning off the UV light and afterglow switching based on **PAM-N1** films obtained by stimuli of NH_3_ vapour and heating. Reproduced with permission from (a) ref. 96 (copyright 2019, Royal Society of Chemistry), (b) ref. 106 (copyright 2018, WILEY-VCH), (c) ref. 110 (Springer Nature Limited) and (d) ref. 109 (copyright 2020, Royal Society of Chemistry).

An encryption algorithm having three different modes of operation has been used to develop information safety applications using URTP materials.^77^ As shown in Fig. 15e, different modes “a”, “b”, and “c” were encrypted by using **ME–IPA**, **MA** and **IPA** (Scheme 2), respectively, and the encryption algorithm enables the real information to be hidden. Besides, the URTP of **MA–IPA** was effectively used to prove personal identity through fingerprints. An inkpad prepared using **MA–IPA** with polyacrylic acid was used to develop the fingerprint on paper (Fig. 15f). Similarly, a 2-dimensional barcode pattern was created by screen-printing the supramolecular framework of **MA–IPA** on filter paper.^91^ The blue-green URTP of **MA–IPA** enabled the information to be identified by scanning the barcode in darkness. Similarly, the distinctly different lifetime, quantum yield, and robust RTP features of **PBC/CB[6]**, **PCC/CB[6]** (Fig. 8a), and **PYCl/CB[6]** (Scheme 7) complexes were used for triple encoding (Fig. 16a).^96^ The initial colourless pattern turned into a bright green display after excitation at 365 nm due to the high quantum yield of **PYCl/CB[6]**. Interestingly, the difference in lifetime enabled sequential phosphorescence displays of **PYCl/CB[6]**, **PCC/CB[6]**, and **PBC/CB[6]**.

URTP of amorphous **PSS** (Scheme 4) and its on/off switching by water vapour has been used for lifetime-encoding application (Fig. 16b).^106^ The green RTP emission of **PSS** film can be masked by making patterns using water. Since the fluorescence remains intact, no change is observed under UV light, even in the presence of water. However, the pattern “KU” created by the water is not anymore RTP active and can be observed by the naked eye. The reversibility of the water-induced patterns increases the applicability of this method. The excitation-dependent URTP has been used for multilevel information encryption using polymers **PDA** (Scheme 7) and **PDNA** (Fig. 11a).^110^ As shown in Fig. 16c, the patterns NTU and RP in the encrypted information “RNTUP” were fabricated using the polymers **PDNA** and **PDA**, respectively, as inks. The initial blue emission under 254 nm irradiation was changed to long-lived blue luminescence to show the false information upon turning off the excitation source. However, after excitation with 365 nm UV light, the correct information of “NTU” was visualized as long-lived yellow emission. Interestingly, the reversibility of encryption has been achieved by erasure using water and regain by thermal treatment. Similarly, the difference in persistent luminescence intensity of **PVA-100-2HC-1** and **PVA-100-P4-1** (Fig. 12a) inks has also been used for anti-counterfeiting applications.^112^

In another attempt, the excellent afterglow properties of **PAM-N1** (Scheme 6) films were found to be advantageous for the detection of volatile solvent vapors (Fig. 16d).^109^ The strong URTP and green afterglow disappeared in the presence of vapours, and at the same time, NH_3_ imparted a drastic emission quenching. The afterglow restoration achieved by thermal removal of NH_3_ ensures an afterglow switch using NH_3_ vapour and temperature. Moreover, the security ink developed using **PAM-N1** was found useful for anti-counterfeiting applications. The flexible and transparent films **T1**@LP and **T2**@LP in **PVA** have been used for relative-humidity sensing and information encryption.^103^ The remarkable RTP characteristics of the hybrid materials generated from aromatic-acid and Al-DMSO matrices have benefited data encryption and decryption applications through allochroic response recognition and optical logic gates.^104^

## 6. Future perspectives

At the moment, crystallization is a prerequisite for small molecule-based organic phosphors to exhibit RTP with a long lifetime and high quantum yield. One of the limitations associated with crystalline assembly is the processability of such materials for use in optoelectronic devices. The continuous search for noncrystalline RTP materials ended up with amorphous RTP materials such as polymers,^116–121^ polymer-supported phosphors,^112–115^ and organic solvent-free liquids.^122^ However, in most of the systems, the amount of optically inactive molecular components used as a support is high, leading to the content of active luminophores being very low. It necessitates new concepts and designs to develop processable luminogens having excellent RTP efficiency.

ORTPs have exhibited many fascinating features useful for imaging and anti-counterfeiting applications. One of the areas that need to be improved is the stimuli-responsive RTP features, which will enable developing tunable emission smart RTP materials. More concrete demonstrations in the field of multi-stimuli-sensitive and dynamic RTP materials are highly required to widen the scope.^123–125^ Another area that can be explored is the nonlinear optical properties of RTP materials, which will bring out newer concepts in demonstrations. Even though a few attempts have been made with lasing^126^ and waveguiding^45^ applications of ORTPs, more detailed studies are envisioned for RTP materials.

ORTPs with emission spanning from blue to orange colours have been mainly reported; however, red-emitting metal-free RTPs are rare. The availability of red or NIR emissive ORTPs will be appropriate to explore in biological applications. However, such a target seems to be the most awaiting one due to the many hurdles associated with ORTPs. Besides, biological applications demand the biocompatibility of organic phosphors, which is another challenge due to the current scarcity of high performing RTPs under physiological conditions. It provides an opportunity to explore further the exciting molecular design of ORTPs suitable for biological applications. In the same line, RTP molecules with two-photon and multi-photon induced emission will also be highly beneficial due to the possible operation of NIR excitation. The recent initial developments on dynamic ORTPs point to a focus on more such candidates.

Recently, Liu and coworkers reported the effect of impurity on the afterglow features of carbazole derivatives.^127^ An isomer present in the commercially available carbazole significantly contributes to RTP. In another report, Chen *et al.* demonstrated that the presence of a trace amount (0.01%) of structurally similar compound 2-(3,4-dimethoxybenzyl)-5-(dimethylamino)isoindoline-1,3-dione formed by the side reaction of an RTP inactive molecule, 5-bromo-2-(3,4-dimethoxybenzyl)isoindoline-1,3-dione, with solvent (DMF) results in strong RTP with *ϕ*_p_ = 25.4% and *τ*_p_ = 48 ms.^128^ The RTP of the impurity is activated by the specific molecular orbital interactions between these two components. It raises serious concerns on the efficiency of organic phosphors. Hence it is advised to check the purity of the samples before analysis.

## 7. Conclusions

In summary, ORTPs have been widely exploited in the last decade due to the advancement in molecular design, control over intermolecular interactions, and deeper fundamental understanding. In this Perspective, we explained the various successful strategies adopted to improve the RTP efficiency of metal-free organic molecules having an exceptionally long RTP lifetime above one second along with high quantum efficiency and remarkable afterglow properties. The major experiments were centered on important aspects such as boosting the population of the triplet excited state through enhanced ISC (*ϕ*_isc_), suppression of nonradiative decay (*k*_nr_) pathways, and slowing down the decay rate of the triplet excited state (*k*_p_). The successful examples pointed to the incorporation of heavy atoms, heteroatoms, and carbonyl groups to improve SOC and control the nonradiative decay of the triplet excitons through crystallization, framework formation, host–guest interactions, and polymer support to obtain efficient RTP candidates. A comparison of the lifetime, quantum yield, and decay parameters revealed that host–guest-based RTPs are better than crystalline, small molecules, and polymer-based RTPs. The most interesting observation is the effective utilization of a long lifetime and the strong afterglow of RTPs in applications spanning from data encryption, anti-counterfeiting, and bioimaging to sensing. Even though many improvised ways to elevate the efficiency of organic phosphors are in place, the real understanding of mechanistic aspects is still missing. Hence this area is expected to make profound revelations shortly through combined experimental and theoretical investigations. The recent increase in both the quality and quantity of publications indicates that RTPs have a bright future. Similarly, the latest developments in this area also point to the vital role of organic functional materials in futuristic applications.

## Conflicts of interest

There are no conflicts to declare.
